# Case-mix-adjusted benchmarking of long-term breast cancer–specific mortality: Insights from a nationwide Swedish registry

**DOI:** 10.1016/j.breast.2026.104835

**Published:** 2026-06-08

**Authors:** Jacob Thurell, Iurii Petrov, Peter Lindgren, Jonas Bergh, Irma Fredriksson, Narsis A. Kiani, Elham Hedayati

**Affiliations:** a Department of Oncology-Pathology, Karolinska Institutet, Anna Steckséns Gata 30A, D2:04, Solna, 171 64, Sweden; b Breast Cancer Center, Department of Breast, Endocrine Tumours and Sarcoma, Karolinska Comprehensive Cancer Center, Karolinska University Hospital, Eugeniavägen 3, Stockholm, 171 76, Sweden; c Department of Microbiology, Tumor- and Cell Biology, Karolinska Institutet, Solnavägen 9, Solna, 171 65, Sweden; d Cancer Centrum Karolinska, Visionsgatan 56b, Stockholm, 171 76, Sweden; e Division of Medical Diagnostics, Radiology, Karolinska University Hospital, C1:46, Stockholm, 141 86, Sweden; f Department of Learning, Informatics, Management and Ethics, Karolinska, Institutet, Tomtebodavägen 18 A, Floor 4, Stockholm, 171 77, Sweden; g The Swedish Institute for Health Economics, Råbygatan 2, Lund, 223 61, Sweden; h Department of Molecular Medicine and Surgery, Karolinska Institutet, Karolinska University Hospital, (L1:00), Stockholm, 171 76, Sweden; i Algorithmic Dynamics Lab, Center for Molecular Medicine, Karolinska Instiutet, Visionsgatan 18, Solna, 171 64, Sweden

**Keywords:** Breast cancer, Cancer survival, Case-mix adjustment, Risk prediction model, Hospital benchmarking, Quality improvement, Population-based study, Long-term outcomes, National cancer registry, Real-world data

## Abstract

**Background:**

Meaningful comparisons of breast cancer (BC) survival across hospitals require appropriate case-mix adjustment. While such adjustment is routine for short-term outcomes, it remains less established for long-term BC-specific mortality (BCSM). We developed case-mix models to evaluate whether robust hospital-level benchmarking of 10-year BCSM can be achieved using routinely collected registry data.

**Methods:**

Using the nationwide Swedish BC registry linked to population registers, we developed full and reduced logistic regression models for 10-year BCSM. The full model included age, tumour, socioeconomic, and comorbidity variables while the reduced model used routinely collected age and tumour variables. Model performance was assessed via discrimination and calibration. The model impact on hospital benchmarking was evaluated through observed-to-expected mortality ratios at the hospital level, comparing expected mortality estimates and shifts in outlier status.

**Results:**

The study included 10,888 patients. Age, stage, and grade were the strongest predictors, while comorbidity and socioeconomic variables added limited incremental predictive value. Both the full and reduced 10-year BCSM models demonstrated similar discrimination and near-perfect calibration. Furthermore, the full and reduced models yielded highly comparable expected mortality rates across 12 hospitals (mean absolute difference 0.6 percentage points), with consistent identification of hospital outliers in all but one hospital.

**Conclusions:**

Routinely collected data on age and tumour characteristics appear to offer a feasible foundation for fair benchmarking of long-term BCSM in a publicly-funded healthcare system. While external validation in contemporary and international cohorts is an important next step, these findings might be relevant to other countries with strong cancer registry infrastructure.

## Highlights

• Case-mix–adjusted hospital benchmarking of long-term breast cancer survival is rare.
• We developed scalable models for hospital-level survival benchmarking.
• Routinely collected registry data appeared sufficient for fair hospital comparisons.
• External validation in a contemporary cohort remains an important next step.

## 1 Introduction

Monitoring long-term survival is critical to evaluating the quality of breast cancer (BC) care, yet such outcomes are rarely compared between hospitals for quality improvement. Our recent scoping review [1] of European cancer benchmarking practices demonstrated widespread use of hospital benchmarking for short-term outcomes, such as 30-day postoperative mortality, but identified few studies assessing long-term BC survival with adjustment for patient case-mix.

Meaningful comparisons require appropriate case-mix adjustment, as crude comparisons risk reflecting differences in patient composition rather than care quality [2]. Tumour factors—particularly stage, subtype, and histological grade—are the primary determinants of breast cancer-specific mortality (BCSM). However, prognosis is also influenced by comorbidity burden, and socioeconomic status (SES). While comorbidity burden affect treatment decisions, tolerance and operability, SES factors (e.g. level of education) impact healthcare-seeking behaviour and treatment adherence. High-quality BC registries are established in several European countries such as the Netherlands, the UK, and the Nordic countries. They typically capture patient-, tumour- and treatment characteristics, but often lack detailed SES and comorbidity data required for comprehensive adjustment without complex data linkage.

Although SES and comorbidity undeniably are important for individual prognosis, their incremental value for hospital-level case-mix adjustment—beyond age and tumour characteristics—remains unclear. Sweden's National Quality Register for Breast Cancer (NKBC) [3] provides an ideal setting to address this question, as it allows for nationwide clinical data to be linked with population-based registers containing individual-level socioeconomic and comorbidity information.

In this study, we investigated whether reliable hospital benchmarking of 10-year BCSM can be achieved using routinely collected tumour variables and age, or whether additional information on SES and comorbidity is required for fair comparisons. To address this, we developed two case-mix models: a comprehensive model including age, tumour, socioeconomic, and comorbidity variables, and a reduced model based on routinely available age and tumour variables. We then compared their performance in individual risk prediction and evaluated their impact on hospital benchmarking by assessing agreement in observed-to-expected (O/E) mortality ratios. By doing so, we aimed to assess the feasibility of using existing cancer registry data for robust hospital-level benchmarking of long-term BCSM, with potential relevance for other European settings with similar data infrastructures.

## 2 Materials and methods

### 2.1 Study design and data source

This population-based cohort study used data from Breast Cancer Database Sweden (BCBaSe 3.0) [4], which includes individuals diagnosed with BC in Sweden between 2008 and 2019. BCBaSe 3.0 is based on linkage between the NKBC and several national demographic and healthcare registers (Supplementary Fig. 1) and includes 114,290 BC cases. The NKBC contains information on patient characteristics, lead-times, diagnostics, tumour characteristics, treatments, and outcomes, reported by the handling breast units. Reporting is not mandatory, yet yearly cross-linking to the mandatory Swedish Cancer Register have shown high coverage (>99%) [5]. Patients are informed in writing about registration and the possibility to opt out at any time, by contacting their treating BC clinic. Ethical approval was obtained from the Swedish Ethical Review Authority (dnr 2021-05189), and the study was registered at ISRCTN (ISRCTN69229101) [6].

### 2.2 Participants

All cases aged 18 years or older at diagnosis of invasive BC stage I–III between 2008 and 2009 were included. This diagnosis periods were chosen as complete data on cause of death were available through December 31, 2019. Patients with stage IV disease at, or within three months after diagnosis, were excluded, as we intended to benchmark survival in BC primarily treated with a curative intent. We therefore also excluded cases that did not undergo surgery. Cases with BC in situ were also excluded.

### 2.3 Outcomes

The primary outcome was 10-year BCSM. BCSM was defined as death attributed to BC in the Swedish Cause of Death Register, either as the underlying or contributing cause. Previous validation studies have shown high accuracy of cause-of-death coding for malignant neoplasms in Sweden [7]. In the primary analyses, deaths from other causes were treated as non-events.

### 2.4 Case-mix variables

The BCBaSe 3.0 database contains approximately 700 variables, from which candidate variables were selected based on clinical expertise and prior research.

Demographic and patient characteristics included **age at diagnosis** and **comorbidity burden** according to the Charlson Comorbidity Index (CCI) [8]. For CCI, an assessment window of six years prior to diagnosis has been suggested as optimal [9], and we chose to include diagnosis up to seven years before BC diagnosis date. SES variables included highest **level of education**, **country of birth**, and **net household income** in the year prior to BC diagnosis. Tumour factors included **T-stage** and **N-stage** according to the TNM classification [10], **estrogen receptor (ER)-** and **progesterone receptor (PR) status, HER2 status**, and **grade (I–III)** [11]. Definitions of all case-mix variables are available in Supplementary Table 1.

Treatment variables were not included in the case-mix models, as this is the part of the care process being evaluated in hospital benchmarking.

### 2.5 Statistical approach

Two fixed-effects logistic regression models were developed: a full model including all case-mix variables, and a reduced model based solely on age and tumour characteristics at baseline.

Logistic regression was used to directly estimate absolute probabilities of BCSM, allowing aggregation of individual predicted probabilities into expected event counts for hospital-level O/E comparisons. This approach provides a transparent and operationally simple framework for benchmarking in registry-based settings [12]. All prespecified variables were retained regardless of statistical significance. Internal validation was performed using bootstrapping (1000 resamples) and an 85/15 train–test split [13]. To account for the potential impact of competing risks, sensitivity analyses were performed using Fine–Gray subdistribution hazard models [14].

Model performance was assessed in terms of discrimination [15] and calibration [16]. Sensitivity analyses were performed across BC subtypes (defined in Supplementary Table 1).

Hospital-level performance was evaluated using O/E ratios for 10-year BCSM among hospitals with ≥30 expected events. Expected deaths were calculated by summing individual predicted probabilities from the full and reduced models. Consistency between models was assessed by: (1) comparing hospital-specific expected BCSM rates between models, (2) calculating Pearson correlation coefficients and visualising agreement using scatter plots, and (3) comparing outlier classification using funnel plots for hospital O/E ratios from both models.

Complete case analyses (CCA) were used as the primary approach to ensure transparency and reproducibility of the benchmarking framework. This approach also reflects how routinely reported registry data would be used in practice for hospital comparisons. As missingness was unlikely to be completely at random, multiple imputation by chained equations (MICE) [17] was performed as sensitivity analysis.

To evaluate the consistency of our approach across different follow-up horizons, we also developed case-mix models for 5-year BCSM following the same statistical approach (Supplementary Tables 7–10; Supplementary Figs. 5–10).

Analyses were conducted in RStudio v.2024.12.1.

## 3 Results

**Fig. 1:**
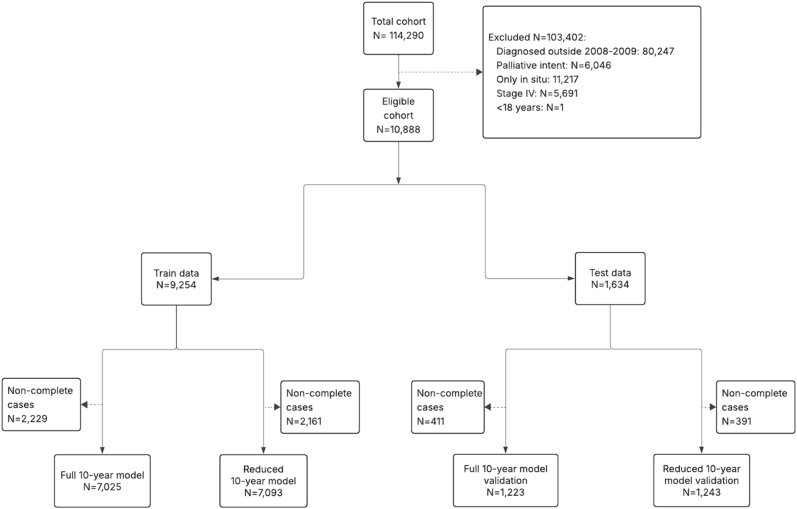
Flowchart of included patients.

Through BCBaSe 3.0, 114,290 cases of primary BC were identified. After exclusions, 10,888 patients remained in the eligible cohort. This cohort was randomly split into a train data set (n = 9254) and a test data set (n = 1634). We used CCA resulting in slightly different sample sizes between the full and the reduced 10-year models. 77% of the eligible cases had complete data on all case-mix variables and were used in the model development (Fig. 1).

A majority of the cases were aged ≥56 years (72%), had more than nine years of education (71%), and were born in Sweden (87%). 61% had no clinically relevant comorbidities. Most tumours were early stage (T1: 56%, node-negative: 66%), and hormone receptor positive (ER+: 85%, PR+: 70%) (Table 1). Approximately 75% of tumours were of luminal subtypes, 13% HER2-positive, and 10% triple-negative (Supplementary Table 2). Subtype classification was incomplete in approximately 15% of cases due to missing data in subtype-defining variables. At hospital level, comorbidity burden showed the greatest variation, with the proportion of patients with CCI ≥2 was 31% in the total cohort but ranged from 14% (Hospital J) to 73% (Hospital H), on the hospital-level. In contrast, SES variables were more evenly distributed across hospitals, although some variation was observed, particularly for level of education (tertiary education ranging from 22% to 40%) (Supplementary Table 3).

**Table 1:** Description of the 10-year cohort.

	10-year cohort N (%)
**Total**	10,888
**Age at BC diagnosis,***years*	
< 45	1002 (9.2)
45–55	2012 (18.5)
56-65	2919 (26.8)
66-75	2732 (25.1)
>75	2223 (20.4)
**Level of education**	
Primary	3100 (28.8)
Secondary	4294 (39.9)
Tertiary	3361 (31.3)
Missing	133 (1.2)
**Household net income**	
1st quartile (lowest)	3303 (30.4)
2nd quartile	3044 (28.0)
3rd quartile	2782 (25.6)
4th quartile	1738 (16.0)
Missing	21 (0.19)
**Country of birth, grouped**	
Sweden	9465 (87.0)
Nordics	592 (5.4)
EU25	342 (3.1)
Rest of the world	480 (4.4)
Missing	9 (0.083)
**Charlson comorbidity burden^1^**	
0	6613 (60.7)
1	885 (8.1)
≥2	3390 (31.1)
**ER-status**	
Positive	8789 (85.0)
Negative	1555 (15.0)
Missing	544 (5.0)
**PR-status**	
Positive	7250 (70.1)
Negative	3086 (29.9)
Missing	552 (5.1)
**HER2-status**	
Positive	1223 (13.3)
Negative	7992 (86.7)
Missing	1673 (15.4)
**Histological grade**	
I	2101 (21.1)
II	4981 (50.1)
III	2869 (28.8)
Missing	937 (8.6)
**T-stage**	
0,1	6141 (56.4)
2	4101 (37.7)
3,4	642 (5.9)
Missing	4 (0.037)
**N-stage**	
0	6870 (66.2)
1	2505 (24.1)
2	692 (6.7)
3	315 (3.0)
Missing	506 (4.6)

Age, tumour stage, and grade were the strongest predictors in both the full and the reduced models. Assessing the additional variables in the full model, comorbidity burden and net household income had the largest impact on 10-year BCSM (Table 2).

**Table 2:** 10-year multivariable logistic regression for the full model (left) and the reduced model (right).

Variable	10-year BCSM	
	**Full model** Odds ratios	**Reduced model** Odds ratios
**Age**		
< 45	0.67*	0.44***
45–55	0.43***	0.29***
56-65	0.49***	0.35***
66-75	0.72**	0.58***
>75	REF	REF
**ER-status**		
Positive	REF	REF
Negative	1.17	1.20
**PR-status**		
Positive	REF	REF
Negative	1.61***	1.59***
**HER2-status**		
Positive	REF	REF
Negative	1.09	1.09
**Histological grade**		
1	REF	REF
2	1.75***	1.75***
3	2.61***	2.55***
**T-stage**		
0,1	REF	REF
2	1.61***	1.60***
3,4	1.99***	2.03***
**N-stage**		
0	REF	REF
1	1.95***	1.96***
2	4.02***	3.98***
3	11.5***	11.3***
**Level of education**		
Primary	REF	-
Secondary	0.99	-
Tertiary	0.96	-
**Net household income**		
Q1 (Lowest)	REF	-
Q2	0.78*	-
Q3	0.63***	-
Q4 (Highest)	0.64***	-
Charlson comorbidity burden*		
0	REF	-
1	1.78***	-
≥2	1.53***	-
**Region of birth**		
Sweden	REF	-
Nordics	1.11	-
EU25	0.89	-
Rest of the world	0.64*	-

*p* < 0.05; **p < 0.01; ***p < 0.001.

**Fig. 2:**
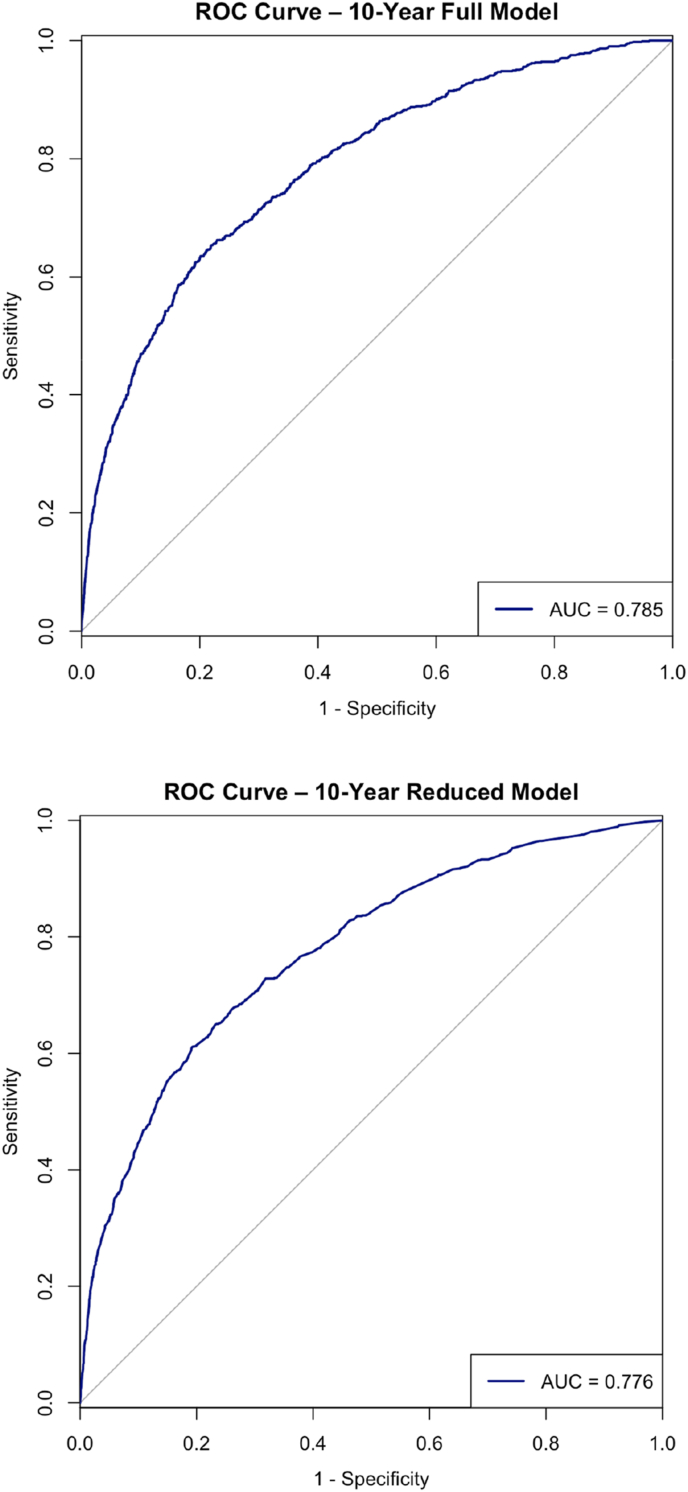
AUC plots of the full versus reduced 10-year BCSM models.

**Fig. 3:**
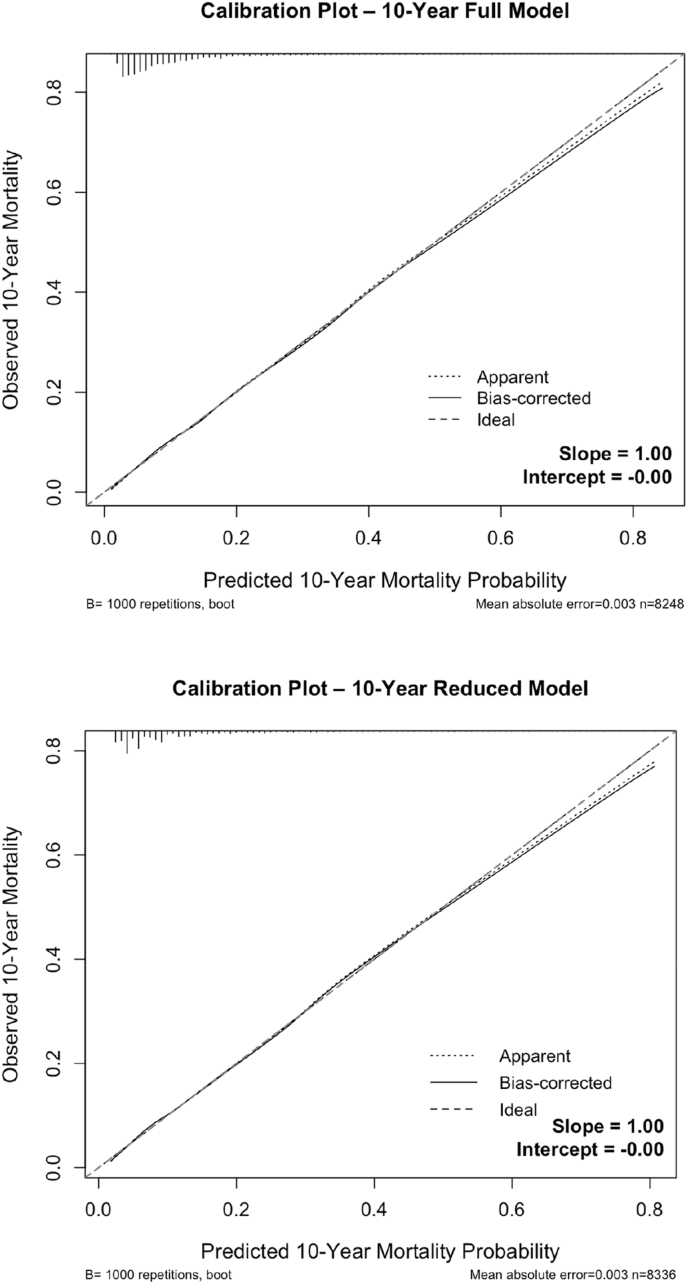
Calibration plots of the full versus reduced 10-year BCSM models.

The full and the reduced models showed very similar performance for individual prediction. Discrimination was comparable across models (C-index 0.79 vs 0.78) and stable across bootstrapped samples, indicating minimal overfitting (Table 3, Fig. 2). Calibration metrics were near-identical, with slopes of 1.00, intercepts of 0.00, demonstrating that the models neither over- nor underestimated risk at any level. Predicted probabilities closely matched observed outcomes across the whole risk spectrum with mean absolute errors of 0.2% (Fig. 3), indicating a reliable basis for calculating expected mortality and subsequent hospital comparisons. No meaningful differences were observed between the full and the reduced models for any performance metric. The 5-year BCSM models were similarly comparable across all performance metrics (Supplementary Table 9, Supplementary Figs. 6–7).

**Table 3:** C-index for the 10-year BCSM models (95% confidence interval).

	10 Y BCSM	
	Full model	Reduced model
Training set	0.785 (0.768-0.802)	0.776 (0.759-0.794)
Test set	0.784 (0.748-0.820)	0.818 (0.783-0.853)
Bootstrapped	0.788 (0.771-0.806)	0.778 (0.760-0.795)

**Fig. 4:**
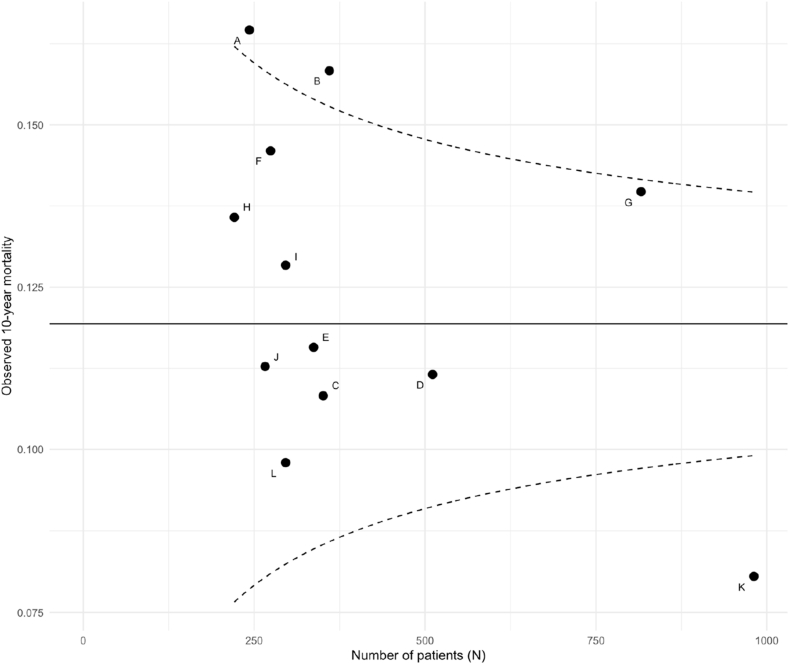
Unadjusted hospital variation in 10-year BCSM with 95 % control limits.

**Fig. 5:**
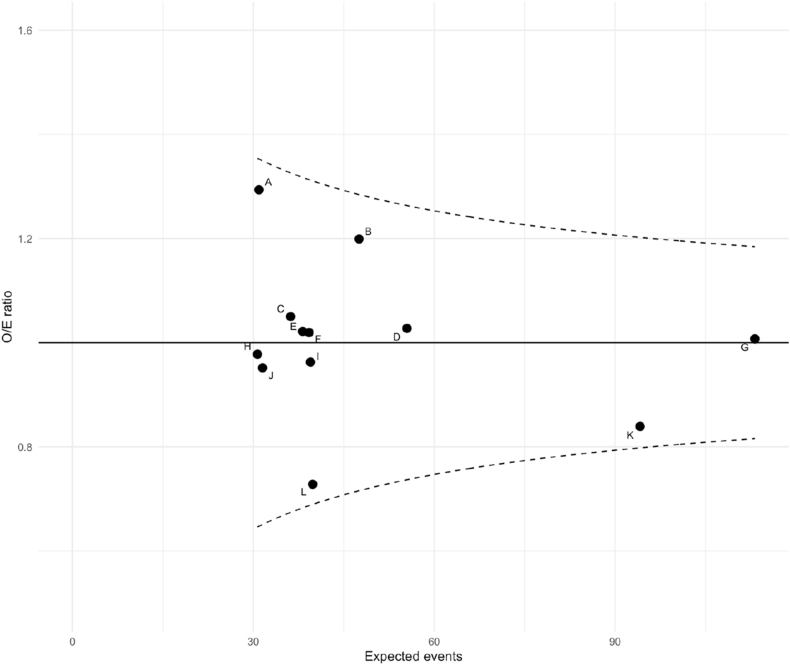
Adjusted hospital variation in 10-year BCSM (Full model) with 95% control limits.

**Fig. 6:**
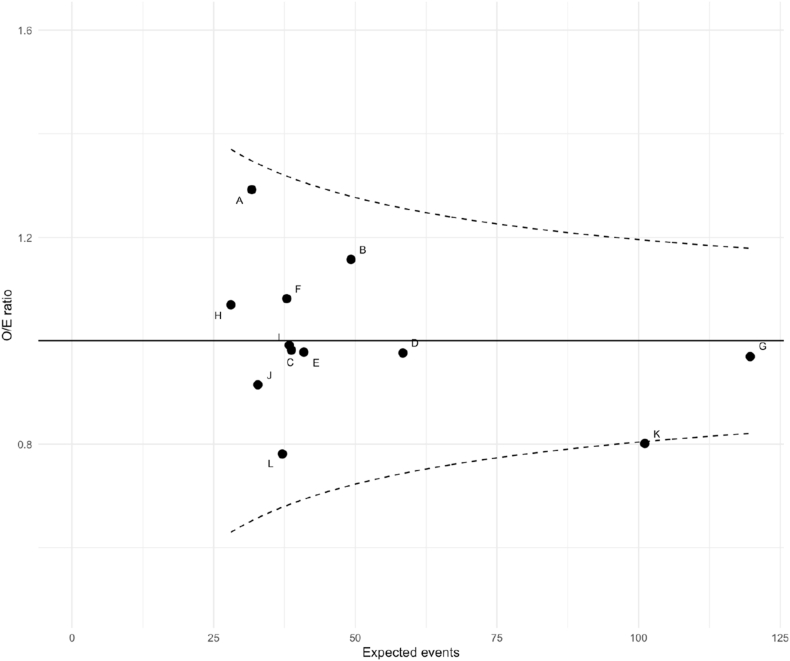
Adjusted hospital variation in 10-year BCSM (Reduced model) with 95% control limits.

We assessed benchmarking consistency by comparing 10-year BCSM O/E ratios for hospitals with at least 30 predicted events. Twelve hospitals met this inclusion criterion. Estimated O/E ratios ranged from 0.73 to 1.29 for the full model and 0.78–1.29 for the reduced model. The mean absolute difference in predicted 10-year BCSM rate was 0.6 percentage points with a maximum difference of 1.2 percentage points (Hospital H: *E*_*full*_ = 13.9% vs *E*_*reduced*_ = 12.7%) (Table 4). The scatterplot confirmed high agreement between models for predicted O/E ratios with negligible systematic bias (Pearson's correlation: 0.93, bias 0.007; Supplementary Fig. 2). The unadjusted funnel plot revealed two negative outliers with higher than average 10-year BCSM (Hospitals A and B) and one positive outlier (Hospital K) (Fig. 4). In the adjusted funnel plots, both negative outliers (A and B) moved within control limits. Hospital K remained a borderline positive outlier in the reduced model (O/E ratio 0.80, 95% CI 0.63–0.98) but moved within control limits in the full model (O/E ratio 0.84, 95% CI 0.66–1.03), representing the only discrepancy in outlier status (Fig. 5, Fig. 6). Overall, there was high concordance between the full and reduced models in both estimated risks and hospital outlier status. The 5-year BCSM models had similarly small model differences in hospital-level expected rates (ranging between 0.2 and 0.7 percentage points) (Supplementary Table 10).

**Table 4:** Absolute difference in hospital level expected 10-year BCSM between the full- and reduced model.

Hospital	Expected full	Expected reduced	Model difference
A	12,7%	12,8%	**−0,1%**
B	13,2%	13,5%	**−0,3%**
C	10,3%	10,9%	**−0,6%**
D	10,9%	11,3%	**−0,4%**
E	11,3%	12,0%	**−0,7%**
F	14,3%	13,6%	**0,7%**
G	13,8%	14,4%	**−0,6%**
H	13,9%	12,7%	**1,2%**
I	13,3%	12,9%	**0,4%**
J	11,8%	12,2%	**−0,4%**
K	9,3%	10,1%	**−0,8%**
L	13,4%	12,4%	**1,0%**

Model performance was consistent across most BC subtypes, with slightly lower discrimination observed in triple-negative disease (C-index 0.71; calibration slope 0.89–0.90; Supplementary Table 4, Supplementary Fig. 3–4). Competing risks models yielded near-identical hospital O/E ratios compared with the logistic models (absolute hospital O/E ratio differences 0.00–0.02; Supplementary Table 5). Missingness was ≤5.1% for all case-mix variables except HER2 status and grade (Table 1). At the hospital level, missingness varied substantially for HER2 status (1.5–56.9%) and histological grade (2.5–13.3%) (Supplementary Table 3). Multiple imputation analyses showed overall comparable O/E ratios as CCA (mean O/E ratio difference MICE vs CCA 0.06 for both full and reduced models). Three hospitals (B, H, and J) showed shifts of 0.12–0.18 in O/E ratios, and one hospital (L) changed outlier status. These differences were highly concordant between the full and the reduced models (Supplementary Table 6). Overall, the agreement between the full and the reduced models in hospital performance estimates remained consistent across all sensitivity analyses.

## 4 Discussion

We developed case-mix models for 10-year BCSM using nationwide Swedish registry data to support hospital-level benchmarking. The full model incorporated SES and comorbidity, whereas the reduced model was limited to age and tumour-related variables available routinely in most BC quality registers. Although SES and comorbidity were associated with BCSM at the individual level, their additional contribution to hospital-level case-mix adjustment appeared limited in this cohort.

Case-mix models prioritize fair hospital benchmarking rather than optimized individual prediction. Their utility depends on both the prognostic strength of included factors and variable distribution across the populations being compared. Strong outcome predictors in our models, such as N-stage, may contribute less to case-mix adjustment if they are uniformly distributed across hospitals. Conversely, weaker predictors can be essential for fair comparison if unevenly distributed. At the hospital level, expected 10-year BCSM rates were very similar between models (mean difference 0.6 percentage points; maximum 1.2 percentage points), and resulted in the same outlier classification in all but one hospital. Notably, even in Hospital H, where comorbidity burden was substantially higher than average (73% vs 31% with ≥2 comorbidities), the difference in expected BCSM remained limited (1.2 percentage points). These results indicate that tumour variables and age yielded highly similar hospital performance estimates compared with the more data-intensive full model in our cohort. Similar findings were observed in the corresponding 5-year analyses, suggesting that the reduced model was robust across time horizons. Although 10-year BCSM is likely the more clinically informative endpoint in BC, 5-year outcomes may allow earlier evaluation of hospital performance.

Age, stage, and grade were the strongest determinants of BCSM, whereas comorbidity and SES showed more moderate associations with outcome. Low SES has been linked to more advanced stage at diagnosis [18], partly through differences in screening uptake and healthcare-seeking behaviour [19]. Low SES has also been linked to lower endocrine treatment adherence [20], but a Swedish study conversely reported lower adherence among patients with higher educational level [21], illustrating that the relationship between SES and BC outcomes may be context-dependent. Similarly, comorbidity influence treatment tolerance [21], operability, and systemic treatment decisions. Despite being established prognostic factors at the individual level, SES and comorbidity had limited impact on hospital-level O/E ratios in our setting. This likely reflects both the limited between-hospital variation in these factors and the context of a publicly funded healthcare system with minimal financial barriers to care, where major SES-related access gradients are less pronounced.

Sensitivity analyses showed slightly lower predictive performance, both for full- and reduced models, in the triple-negative subtype, likely reflecting the aggressive and heterogeneous disease course in this group. Competing risks did not meaningfully alter the hospital O/E ratios compared to the main logistic models, indicating that transparent and easily interpretable logistic models were sufficient for robust benchmarking in this cohort. Multiple imputation yielded O/E ratios comparable to the CCA for most hospitals. However, three hospitals showed larger and consistent deviations across models (0.12–0.18), suggesting sensitivity to missing data patterns in specific settings rather than systematic differences across all hospitals. The study period represents an early phase of nationwide registry reporting, when data completeness was lower than in more recent cohorts, indicating that the impact of missing data on benchmarking is likely smaller in contemporary data. Overall, CCA appears adequate for population-level benchmarking in this setting, but multiple imputation remains an important robustness check in registry-based studies.

Unadjusted 10-year BCSM comparisons identified three apparent outliers—two with higher and one with lower mortality than the national average. After case-mix adjustment, all hospitals were within the 95% control limits, highlighting the importance of risk adjustment for fair benchmarking. Adjusted 10-year BCSM O/E ratios ranged from 0.73 to 1.29, with most hospitals (8/12) falling between 0.9 and 1.1, indicating broadly similar outcomes across high-volume centres during the study period. However, the study was underpowered to detect moderate hospital outliers, as the threshold of 30 predicted events resulted in relatively wide 95% control limits (approximately 0.65–1.35). Given Sweden's population size, aggregation of multiple calendar years will likely remain necessary to detect more moderate survival deviations in contemporary cohorts. To expand on these benchmarking efforts, future studies with more granular treatment and care process data, including treatment intensity, completion, adherence, and other longitudinal aspects of care delivery, will be important to better understand how differences in care contribute to hospital-level survival variation.

Few European studies have benchmarked BC survival at hospital level. One Swedish study by our group [22] benchmarked survival in HER2-positive early BC between two Swedish regions. This study showed the importance of case-mix adjustment but was limited to one BC subtype and did not develop case-mix models for routine use. A UK study provided regional case-mix–adjusted survival estimates following BC diagnosis [23], although their primary aim was to compare chemotherapy use between the providers. They found small to modest regional survival disparities, which is in line with our findings. Pfister and colleagues [24] investigated whether fair risk adjustment of cancer survival for benchmarking could be achieved without stage information. Their aim was related to ours, assessing whether a more parsimonious model would yield comparable conclusions regarding hospital performance. They found that stage contributed little additional value for benchmarking once information on comorbidity and socioeconomic factors was included. Taken together, their findings, together with ours, suggest that while a broad set of variables may improve individual-level prediction of cancer mortality, not all variables are necessarily required for reliable hospital-level benchmarking.

A major strength of this study is the use of a nationwide, population-based cohort with high completeness, providing reliable data on long-term BC outcomes. Several limitations should be noted. Follow-up ended in 2019, so results do not reflect recent therapeutic advances, such as the widespread introduction of newer targeted therapies. However, excluding the COVID-19 pandemic could provide a more stable baseline for model development, as there were temporary shifts in both care processes and death patterns during the pandemic. Nonetheless, external validation in contemporary cohorts and across other European countries with high-quality BC registries is a critical next step before real-world implementation. In the fast-evolving field of BC treatment, calibration drift [25] over time may occur, which necessitates regular updates and re-calibration of the model. Data on performance status, BMI, and smoking were unavailable, which are potential sources of residual confounding. Potential misclassification in the Cause of Death Register may also have influenced results.

To our knowledge, this is among the first European studies to develop and internally validate case-mix models for real-world benchmarking of long-term BCSM. Our study addresses a common challenge shared by many European countries: the lack of socioeconomic and comorbidity data in national cancer registries. While these factors are important for individual-level prediction, our findings suggest that routinely collected tumour variables and age may be sufficient for fair benchmarking of long-term BCSM in a publicly funded healthcare system – with potential relevance to other European countries with similar registry structures.

## Declaration of generative AI and AI-assisted technologies in the writing process

During the preparation of this work, the authors used *ChatGPT* and Google Gemini to optimise the R code for the statistical analyses and enhance the fluency and academic tone of the manuscript. After using these tools, the authors checked all code, reviewed and edited the content as needed and take full responsibility for the content of the publication.

## Role of funding source

The funder of the study had no role in study design, data collection, data analysis, data interpretation, or writing of the report.

## CRediT authorship contribution statement

**Jacob Thurell:** Conceptualization, Data curation, Formal analysis, Methodology, Writing – original draft, Writing – review & editing. **Iurii Petrov:** Conceptualization, Data curation, Formal analysis, Methodology, Validation, Writing – review & editing. **Peter Lindgren:** Conceptualization, Methodology, Supervision, Writing – review & editing. **Jonas Bergh:** Conceptualization, Methodology, Supervision, Writing – review & editing. **Irma Fredriksson:** Conceptualization, Methodology, Resources, Supervision, Writing – review & editing. **Narsis A. Kiani:** Conceptualization, Methodology, Supervision, Writing – review & editing. **Elham Hedayati:** Conceptualization, Funding acquisition, Methodology, Project administration, Supervision, Writing – review & editing.

## Declaration of competing interest

J.T. report that this work was funded by the Innovative Health Initiative through the project H2O. J.T. also received funding from Radiumhemmets forskningsfonder. All payments to the Karolinska University Hospital.

I.P. declares being a member of the AI committee of Cancer Center Karolinska.

P.L. has received institutional grants from Amgen, AstraZeneca, Janssen, MSD, Novartis, PhRMA, and Pfizer, and participates in advisory boards for AstraZeneca, Takeda, and MSD. All payments were made to the institution.

J.B. has received research grants from Amgen, AstraZeneca, Bayer, Merck, Pfizer, Roche, and Sanofi-Aventis for molecular marker and clinical studies; all payments were to Karolinska Institutet and/or Karolinska University Hospital. J.B. has also received honoraria from Novartis, Roche, and AstraZeneca for chairing meetings or delivering lectures. J.B. has consulted for Stratipath AB, and received payments to Coronis and Asklepios Cancer Research AB. J.B. holds stocks in Stratipath AB, a company involved in AI-based diagnostics for breast cancer. J.B. co-authored a chapter on “Prognostic and Predictive factors in early, non-metastatic breast cancer” in *UpToDate* (honoraria to Asklepios Medicin HB).

I.F. has received research funding for this manuscript from the Näsström donation to Karolinska Institutet. Has also received an institutional grant from MSD for a research project performed under the Master Collaboration Agreement between MSD and Karolinska Institutet, unrelated to this work. Has lectured at postgraduate BC courses and Oncology Nurse courses arranged by Astra Zeneca with payment to employer, hour by hour. I.F. is Chairman of The Swedish National Quality Register for Breast Cancer, Board Member of the Swedish Breast Cancer Group and Board Member of the Percy Falck Research Foundation for Breast and Prostatic Cancer.

N.K. declare no conflicts of interest.

E.H. is co-founder and board member of TrueDose AB. She also has an ownership interest including patents in the company.
