# Supplementary material for: Case-mix-adjusted benchmarking of long-term breast cancer–specific mortality: Insights from a nationwide Swedish registry
Source: Breast. 2026 Jun 8;88:104835. doi: 10.1016/j.breast.2026.104835 (PMC13272560; doi:10.1016/j.breast.2026.104835)

**Supplementary Table 1.** Definitions of case-mix variables and breast cancer subtypes

| **Case-mix variables** | **Definition** | **Categories** | **Available in NKBC** |
| --- | --- | --- | --- |
| **Age at diagnosis** | Patient’s age at date of diagnosis | <45, 45-55, 56-65, 66-75,>75 | YES |
| **T-stage** | According to the TNM-classification. Data derived from the pathological report for primary operated cases, and clinical staging in cases treated with neoadjuvant therapy. | 1= T0, T1 2=T2 3=T3, T4 | YES |
| **N-stage** | According to the TNM-classification. Data derived from the pathological report for primary operated cases, and clinical staging in cases treated with neoadjuvant therapy. | 0=N0 1=N1 2=N2 3=N3 | YES |
|  |  |  |  |
|  |  |  |  |
| **ER status** | Considered positive when ≥10% of the epithelial tumour cells showed positive staining. | Positive or negative | YES |
| **PR status** | Considered positive when ≥10% of the epithelial tumour cells showed positive staining. | Positive or negative | YES |
| **HER2 status** | Deemed positive if immunohistochemistry (IHC) was 3+, or 2+ with gene amplification confirmed by in situ hybridisation. | Positive or negative | YES |
| **Histological grade** | Defined according to the Nottingham histological grade (NHG) system. | Grade I, II, or III | YES |
| **Comorbidity burden** | Defined as the patient’s score on the Charlson comorbidity index, applying diagnoses existing up to 7 years before calendar date of breast cancer diagnosis. | 0, 1, and ≥2 | NO |
| **Net household income** | Annual net income of the patient’s household the year prior to breast cancer diagnosis calendar year. | Categorized into quartiles based on the whole eligible cohort. | NO |
| **Level of education** | Patient’s highest attained level of education. | Primary (≤9 years), secondary (10-12 years), and tertiary (≥12 years) | NO |
| **Country of birth** | Patient’s country of birth. | Grouped into “Sweden”, “Nordics except Sweden”, “EU25 except Nordics”, and “Rest of the world”. | NO |
| **BC subtypes** | **Definition** |  |  |
| **Luminal BC (all)** | All cases that were ER-positive and HER2-negative |  |  |
| **Luminal A** | All luminal cases with histological grade I or grade II and KI-67<20 % |  |  |
| **Luminal B** | All luminal cases with histological grade III or grade II and KI-67≥20 % |  |  |
| **HER2-positive BC** | All cases that were HER2-positive |  |  |
| **Triple-negative BC** | All cases that were ER-negative, PR-negative and HER2-negative |  |  |

**Supplementary Table 2.** Distribution of breast cancer subtypes in the 5- and 10-year cohort

| **Biological subtype** | **5-year cohort N (%)** | **10-year cohort N (%)** |
| --- | --- | --- |
| **Total** | 44,638 | 10,888 |
| **Luminal (all)** |  |  |
| Yes | 31,312 (77.7) | 6,992 (76.1) |
| No | 9,032 (22.3) | 2,195 (23.9) |
| Not classifiable | 4,301 (9.6) | 1,701 (15.6) |
| **Luminal A^1^** |  |  |
| Yes | 13,040 (37.4) | 2,320 (29.8) |
| No | 21,805 (62.6) | 5,467 (70.2) |
| Not classifiable | 9,793 (21.9) | 3,101 (28.5) |
| **Luminal B^1^** |  |  |
| Yes | 8,087 (23.2) | 1,266 (16.3) |
| No | 26,758 (76.8) | 6,521 (83.7) |
| Not classifiable | 9,793 (21.9) | 3,101 (28.5) |
| **HER2-positive** |  |  |
| Yes | 5,300 (13.1) | 1,223 (13.3) |
| No | 35,122 (86.9) | 7,992 (86.7) |
| Not classifiable | 4,216 (9.4) | 1,673 (15.4) |
| **Triple-negative** |  |  |
| Yes | 3,545 (8.8) | 939 (10.2) |
| No | 36,753 (91.2) | 8,239 (89.8) |
| Not classifiable | 4,340 (9.7) | 1,710 (15.7) |
| ^1^A high fraction of the cases before Jan, 2013 had no registered KI-67. These cases were not classified into Luminal A or B if histological grade II. | | |

**S**

**Supplementary Table 3:** 10-year BCSM Hospital distribution of case-mix variables and level of missingness

| **Hospital** | A | B | C | D | E | F | G | H | I | J | K | L |
| --- | --- | --- | --- | --- | --- | --- | --- | --- | --- | --- | --- | --- |
| **Age at diagnosis** |  |  |  |  |  |  |  |  |  |  |  |  |
| <45 years | 10% | 11% | 8% | 10% | 8% | 7% | 12% | 8% | 6% | 10% | 13% | 9% |
| 45-55 years | 21% | 21% | 20% | 23% | 18% | 19% | 18% | 19% | 18% | 16% | 21% | 17% |
| 56-65 years | 24% | 29% | 31% | 30% | 28% | 25% | 26% | 32% | 29% | 25% | 24% | 25% |
| 66-75 years | 27% | 21% | 22% | 20% | 26% | 26% | 24% | 24% | 27% | 27% | 23% | 22% |
| >75 years | 18% | 19% | 19% | 18% | 21% | 24% | 20% | 17% | 20% | 23% | 19% | 27% |
| **Level of education** |  |  |  |  |  |  |  |  |  |  |  |  |
| Primary | 29% | 24% | 21% | 23% | 30% | 31% | 29% | 23% | 34% | 29% | 20% | 38% |
| Secondary | 40% | 44% | 40% | 38% | 37% | 38% | 37% | 43% | 44% | 47% | 37% | 40% |
| Tertiary | 31% | 33% | 39% | 40% | 33% | 31% | 35% | 34% | 22% | 24% | 44% | 22% |
| Missing | 1,07% | 1,48% | 1,18% | 0,66% | 1,43% | 1,33% | 1,83% | 0,35% | 1,12% | 0,94% | 1,79% | 0,59% |
| **Net household income** |  |  |  |  |  |  |  |  |  |  |  |  |
| Q1 (Lowest) | 31% | 27% | 26% | 28% | 29% | 34% | 30% | 25% | 36% | 33% | 27% | 31% |
| Q2 | 28% | 28% | 27% | 27% | 27% | 29% | 29% | 32% | 29% | 29% | 27% | 30% |
| Q3 | 26% | 27% | 24% | 27% | 28% | 26% | 25% | 31% | 25% | 25% | 23% | 21% |
| Q4 (Highest) | 15% | 18% | 24% | 18% | 16% | 11% | 17% | 13% | 11% | 13% | 23% | 17% |
| Missing | 0,00% | 0,25% | 0,47% | 0,16% | 0,12% | 0,33% | 0,41% | 0,00% | 0,28% | 0,00% | 0,21% | 0,00% |
| **Country of birth** |  |  |  |  |  |  |  |  |  |  |  |  |
| Sweden | 91% | 87% | 85% | 82% | 85% | 89% | 84% | 94% | 92% | 92% | 78% | 86% |
| Nordics | 5% | 4% | 8% | 6% | 4% | 3% | 4% | 4% | 3% | 6% | 9% | 8% |
| EU25 | 2% | 2% | 4% | 5% | 5% | 3% | 4% | 1% | 3% | 1% | 5% | 3% |
| Rest of the world | 3% | 6% | 4% | 7% | 7% | 5% | 8% | 2% | 2% | 1% | 8% | 4% |
| Missing | 0,36% | 0,00% | 0,24% | 0,16% | 0,00% | 0,00% | 0,20% | 0,00% | 0,00% | 0,00% | 0,07% | 0,00% |
| **Charlson comorbidity score** |  |  |  |  |  |  |  |  |  |  |  |  |
| 0 | 67% | 67% | 72% | 70% | 65% | 49% | 69% | 23% | 58% | 76% | 70% | 43% |
| 1 | 7% | 11% | 11% | 7% | 9% | 4% | 11% | 4% | 8% | 10% | 9% | 5% |
| ≥2 | 25% | 22% | 18% | 23% | 25% | 47% | 20% | 73% | 33% | 14% | 21% | 52% |
| Missing | 0,00% | 0,00% | 0,00% | 0,00% | 0,00% | 0,00% | 0,00% | 0,00% | 0,00% | 0,00% | 0,00% | 0,00% |
| **ER-status** |  |  |  |  |  |  |  |  |  |  |  |  |
| Positive | 83% | 85% | 85% | 92% | 87% | 88% | 84% | 84% | 84% | 87% | 82% | 89% |
| Negative | 17% | 15% | 15% | 9% | 13% | 12% | 16% | 16% | 16% | 13% | 18% | 11% |
| Missing | 4,63% | 0,74% | 4,47% | 5,58% | 3,46% | 2,99% | 6,63% | 1,40% | 4,19% | 3,76% | 4,72% | 7,37% |
| **PR-status** |  |  |  |  |  |  |  |  |  |  |  |  |
| Positive | 64% | 71% | 67% | 71% | 73% | 74% | 66% | 74% | 74% | 67% | 65% | 69% |
| Negative | 36% | 29% | 33% | 29% | 27% | 26% | 34% | 26% | 26% | 33% | 35% | 31% |
| Missing | 4,63% | 0,99% | 4,71% | 6,08% | 3,34% | 2,99% | 6,42% | 1,40% | 4,47% | 4,08% | 4,79% | 7,37% |
| **HER2-status** |  |  |  |  |  |  |  |  |  |  |  |  |
| Positive | 17% | 12% | 10% | 10% | 25% | 10% | 14% | 17% | 11% | 11% | 13% | 10% |
| Negative | 83% | 88% | 90% | 90% | 75% | 90% | 86% | 83% | 89% | 89% | 87% | 90% |
| Missing | 4,98% | 1,48% | 4,24% | 7,55% | 56,90% | 3,65% | 6,93% | 18,20% | 7,54% | 5,64% | 19,90% | 8,55% |
| **Histological grade** |  |  |  |  |  |  |  |  |  |  |  |  |
| 1 | 16% | 14% | 22% | 17% | 23% | 19% | 18% | 27% | 24% | 16% | 20% | 23% |
| 2 | 46% | 54% | 53% | 50% | 42% | 48% | 54% | 42% | 56% | 58% | 50% | 52% |
| 3 | 37% | 32% | 24% | 33% | 35% | 33% | 28% | 31% | 20% | 26% | 30% | 24% |
| Missing | 4,27% | 3,21% | 9,65% | 11,50% | 6,09% | 4,65% | 9,28% | 2,46% | 6,15% | 8,15% | 13,30% | 7,08% |
| **T-stage** |  |  |  |  |  |  |  |  |  |  |  |  |
| 0,1 | 59% | 49% | 60% | 56% | 56% | 59% | 40% | 56% | 58% | 61% | 58% | 61% |
| 2 | 38% | 45% | 37% | 38% | 40% | 35% | 45% | 40% | 37% | 35% | 36% | 37% |
| 3 | 3% | 7% | 3% | 6% | 4% | 7% | 15% | 5% | 6% | 5% | 6% | 2% |
| Missing | 0,00% | 0,00% | 0,24% | 0,00% | 0,00% | 0,00% | 0,10% | 0,00% | 0,00% | 0,00% | 0,00% | 0,00% |
| **N-stage** |  |  |  |  |  |  |  |  |  |  |  |  |
| 0 | 67% | 62% | 71% | 64% | 62% | 66% | 65% | 63% | 62% | 69% | 68% | 64% |
| 1 | 23% | 26% | 23% | 28% | 26% | 24% | 23% | 24% | 26% | 23% | 25% | 26% |
| 2 | 8% | 7% | 5% | 6% | 9% | 5% | 7% | 8% | 7% | 7% | 5% | 8% |
| 3 | 2% | 5% | 1% | 2% | 4% | 5% | 5% | 4% | 5% | 2% | 1% | 2% |
| Missing | 6,41% | 6,17% | 5,18% | 0,66% | 2,03% | 1,66% | 5,40% | 3,51% | 6,98% | 5,33% | 1,07% | 2,36% |

**Supplementary Table 4.** C-index for the 10-year BCSM models in subgroups

|  | **10 Y BCSM**  **C-index (95 % CI)** |
| --- | --- |
| **Full model** |  |
| Luminal A | 0.756 (0.710-0.803) |
| Luminal B | 0.742 (0.705-0.779) |
| Triple-negative | 0.713 (0.666-0.760) |
| HER2 positive | 0.768 (0.729-0.807) |
| **Reduced model** |  |
| Luminal A | 0.739 (0.687-0.790) |
| Luminal B | 0.725 (0.687-0.764) |
| Triple-negative | 0.712 (0.666-0.758) |
| HER2 positive | 0.757 (0.717-0.798) |

**Supplementary Table 5:** Comparing 10year BCSM O/E ratios per hospital for logistic models versus competing risks (Fine Gray) models.

|  | **Full model O/E** | **Full Fine Gray** | **Difference** | **Reduced model O/E** | **Reduced Fine Gray** | **Difference** |
| --- | --- | --- | --- | --- | --- | --- |
| **HOSPITAL** |  |  |  |  |  |  |
| A | 1,29 | 1,28 | **0,01** | 1,29 | 1,28 | **0,01** |
| B | 1,20 | 1,19 | **0,01** | 1,16 | 1,15 | **0,01** |
| C | 1,05 | 1,03 | **0,02** | 0,98 | 0,96 | **0,02** |
| D | 1,03 | 1,02 | **0,01** | 0,98 | 0,97 | **0,01** |
| E | 1,02 | 1,03 | **-0,01** | 0,98 | 0,98 | **0,00** |
| F | 1,02 | 1,01 | **0,01** | 1,08 | 1,08 | **0,00** |
| G | 1,01 | 0,99 | **0,02** | 0,97 | 0,96 | **0,01** |
| H | 0,98 | 0,98 | **0,00** | 1,07 | 1,07 | **0,00** |
| I | 0,96 | 0,95 | **0,01** | 0,99 | 0,98 | **0,01** |
| J | 0,95 | 0,94 | **0,01** | 0,92 | 0,91 | **0,01** |
| K | 0,84 | 0,83 | **0,01** | 0,80 | 0,79 | **0,01** |
| L | 0,73 | 0,72 | **0,01** | 0,78 | 0,78 | **0,00** |

**Supplementary Table 6:** Comparing 10-year BCSM O/E ratios per hospital for logistic models versus multiple imputation models (MICE).

|  | **Full model O/E** | **Full MICE** | **Difference** | **Reduced model O/E** | **Reduced MICE** | **Difference** |
| --- | --- | --- | --- | --- | --- | --- |
| **HOSPITAL** |  |  |  |  |  |  |
| A | 1,29 | 1,24 | **0,05** | 1,29 | 1,23 | **0,06** |
| B | 1,20 | 1,08 | **0,12** | 1,16 | 1,06 | **0,10** |
| C | 1,05 | 1,07 | **-0,02** | 0,98 | 1,02 | **-0,04** |
| D | 1,03 | 1,04 | **-0,01** | 0,98 | 1,01 | **-0,03** |
| E | 1,02 | 1,03 | **-0,01** | 0,98 | 0,99 | **-0,01** |
| F | 1,02 | 1,02 | **0,00** | 1,08 | 1,06 | **0,02** |
| G | 1,01 | 1,00 | **0,01** | 0,97 | 0,96 | **0,01** |
| H | 0,98 | 0,82 | **0,16** | 1,07 | 0,91 | **0,16** |
| I | 0,96 | 0,97 | **-0,01** | 0,99 | 1,01 | **-0,02** |
| J | 0,95 | 0,77 | **0,18** | 0,92 | 0,74 | **0,18** |
| K | 0,84 | 0,91 | **-0,07** | 0,80 | 0,86 | **-0,06** |
| **L*** | 0,73 | 0,69 | **0,04*** | 0,78 | 0,75 | **0,03** |
| *****Shifting outlier status | | | | | | |

**Supplementary Table 7:** Description of the 5-year BCSM cohort

|  | **5-year cohort N (%)** |
| --- | --- |
| **Total** | 44,638 |
| **Age at BC diagnosis,** *years* |  |
| < 45 | 3,889 (8.7) |
| 45–55 | 8,031 (18.0) |
| 56-65 | 11,141 (25.0) |
| 66-75 | 12,923 (29.0) |
| >75 | 8,645 (19.4) |
| **Level of education** |  |
| Primary | 11,413 (25.9) |
| Secondary | 18,134 (41.1) |
| Tertiary | 14,560 (33.0) |
| Missing | 531 (1.2) |
| **Household net income** |  |
| 1^st^ quartile (lowest) | 12,057 (27.1) |
| 2^nd^ quartile | 11,985 (26.9) |
| 3^rd^ quartile | 11,293 (25.4) |
| 4^th^ quartile | 9,193 (20.6) |
| Missing | 110 (0.25) |
| **Country of birth, grouped** |  |
| Sweden | 38,358 (86.1) |
| Nordics | 2,368 (5.3) |
| EU25 | 1,455 (3.3) |
| Rest of the world | 2,378 (5.3) |
| Missing | 79 (0.18) |
| **Charlson comorbidity burden^1^** |  |
| 0 | 28,206 (63.2) |
| 1 | 4,052 (9.1) |
| ≥2 | 12,380 (27.7) |
| **ER-status** |  |
| Positive | 36,933 (84.4) |
| Negative | 5,813 (13.6) |
| Missing | 1,892 (4.2) |
| **PR-status** |  |
| Positive | 31,185 (73.0) |
| Negative | 11,511 (27.0) |
| Missing | 1,942 (4.4) |
| **HER2-status** |  |
| Positive | 5,300 (13.1) |
| Negative | 35,122 (86.9) |
| Missing | 4,216 (9.4) |
| **Histological grade** |  |
| I | 8,551 (21.2) |
| II | 20,194 (50.1) |
| III | 11,592 (28.7) |
| Missing | 4,301 (9.6) |
| **T-stage** |  |
| 0,1 | 25,806 (57.8) |
| 2 | 16,112 (36.1) |
| 3,4 | 2,707 (6.1) |
| Missing | 13 (0.030) |
| **N-stage** |  |
| 0 | 28,928 (67.9) |
| 1 | 10,128 (23.8) |
| 2 | 2,407 (5.6) |
| 3 | 1,145 (2.7) |
| Missing | 2,030 (4.5) |
| ^1^Grouped score on the Charlson comorbidity index | |

**Supplementary Table 8:** 5-year multivariable logistic regression for the full model (left) and the reduced model (right)

|  | **5-year BCSM** | |
| --- | --- | --- |
| **Variable** | **Full model**  Odds ratios | **Reduced model**  Odds ratios |
| **Age** |  |  |
| < 45 | 0.41*** | 0.26*** |
| 45–55 | 0.33*** | 0.21*** |
| 56-65  66-75  >75 | 0.34*** | 0.24*** |
|  | 0.51*** | 0.42*** |
|  | REF | REF |
| **ER-status** |  |  |
| Positive | REF | REF |
| Negative | 1.88*** | 1.92*** |
| **PR-status** |  |  |
| Positive | REF | REF |
| Negative | 1.68*** | 1.65*** |
| **HER2-status** |  |  |
| Positive | REF | REF |
| Negative | 1.32*** | 1.32*** |
| **Histological grade** |  |  |
| 1 | REF | REF |
| 2 | 1.57*** | 1.55*** |
| 3 | 2.89*** | 2.83*** |
| **T-stage** |  |  |
| 0,1 | REF | REF |
| 2 | 1.83*** | 1.22*** |
| 3,4 | 2.67*** | 2.76*** |
| **N-stage** |  |  |
| 0 | REF | REF |
| 1 | 1.88*** | 1.88*** |
| 2 | 4.28*** | 4.34*** |
| 3 | 8,59*** | 8.64*** |
| **Level of education** |  |  |
| Primary | REF | - |
| Secondary | 0.80*** | - |
| Tertiary | 0.80** | - |
| **Net household income** |  |  |
| Q1 (Lowest) | REF | - |
| Q2 | 0.79*** | - |
| Q3 | 0.67*** | - |
| Q4 (Highest) | 0.60*** | - |
| **Charlson comorbidity burden*** |  |  |
| 0 | REF | - |
| 1 | 1.44*** | - |
| ≥2 | 1.55*** | - |
| **Region of birth** |  |  |
| Sweden | REF | - |
| Nordics | 1.02 | - |
| EU25 | 0.75 | - |
| Rest of the world | 0.68** | - |

**Supplementary Table 9:** C-index for the 5-year BCSM models (95 % confidence interval)

|  | **5 Y BCSM** | |
| --- | --- | --- |
|  | **Full model** | **Reduced model** |
| Training set | 0.840 (0.830–0.849) | 0.830 (0.820-0.840) |
| Test set | 0.829 (0.806-0.852) | 0.819 (0.795-0.844) |
| Bootstrapped | 0.840 (0.832-0.849) | 0.830 (0.820-0.839) |

**Supplementary Table 10.** Absolute difference in hospital level expected 5-year BCSM between the full- and reduced model

| **Hospital** | **Expected full** | **Expected reduced** | **Model difference** |
| --- | --- | --- | --- |
| A | 6.1% | 6.3% | **-0,2%** |
| B | 6.2% | 6.5% | **-0,3%** |
| C | 5.0% | 5.5% | **-0,5%** |
| D | 4.7% | 5.0% | **-0,3%** |
| E | 5.6% | 5.9% | **-0,3%** |
| F | 7.9% | 7.2% | **0,7%** |
| G | 6.8% | 7.2% | **-0,4%** |
| H | 6.3% | 5.7% | **0.6%** |
| I | 7.1% | 6.6% | **0,5%** |
| J | 5.9% | 5.7% | **0,2%** |
| K | 4.8% | 5.3% | **-0,5%** |
| L | 7.3% | 6.6% | **0.7%** |

**Figures**

**Figure 1.** Flowchart of included patients

**
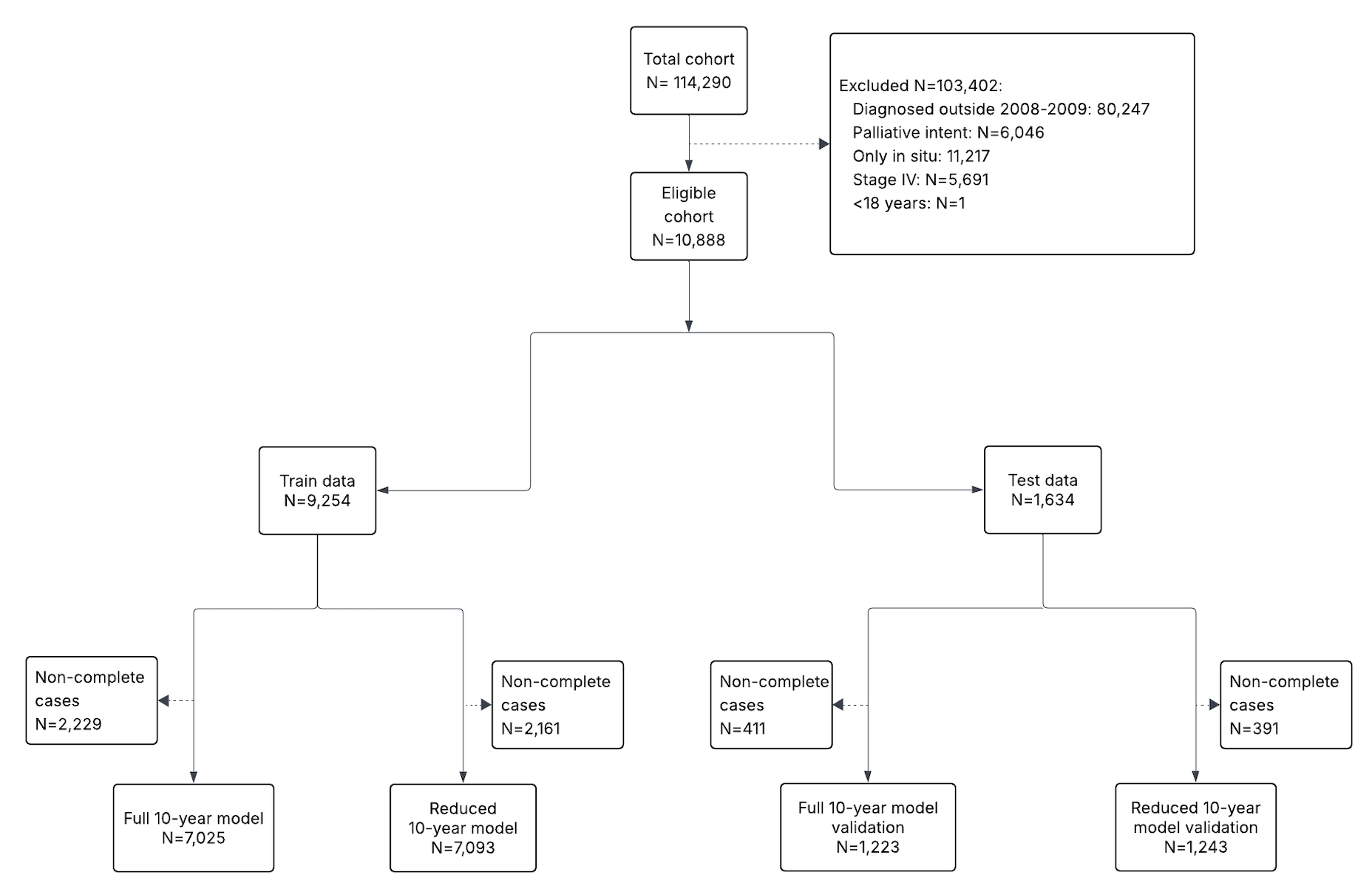
**

**Figure 2:** AUC plots of the full versus reduced 10-year BCSM models

**
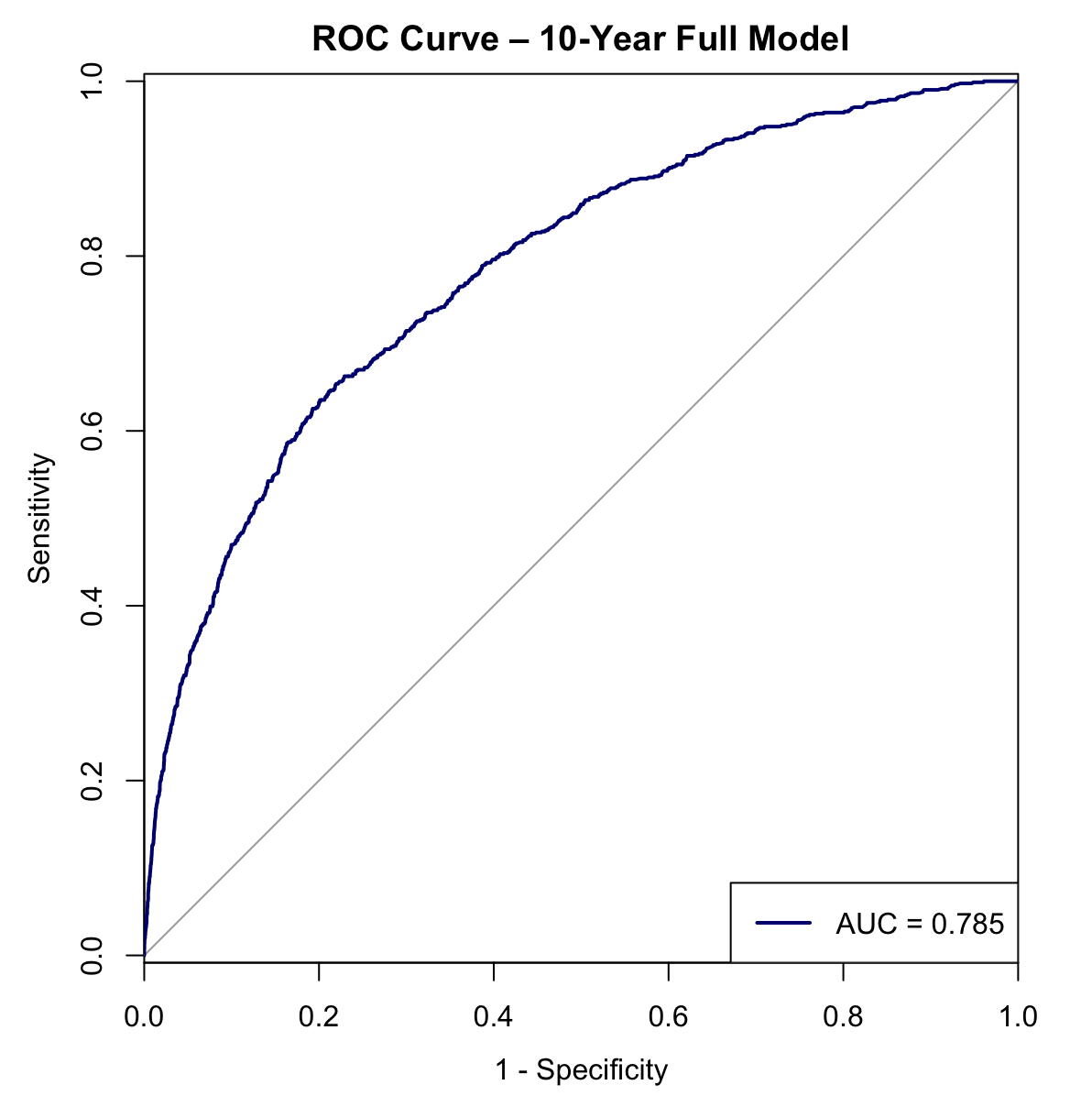
**

**
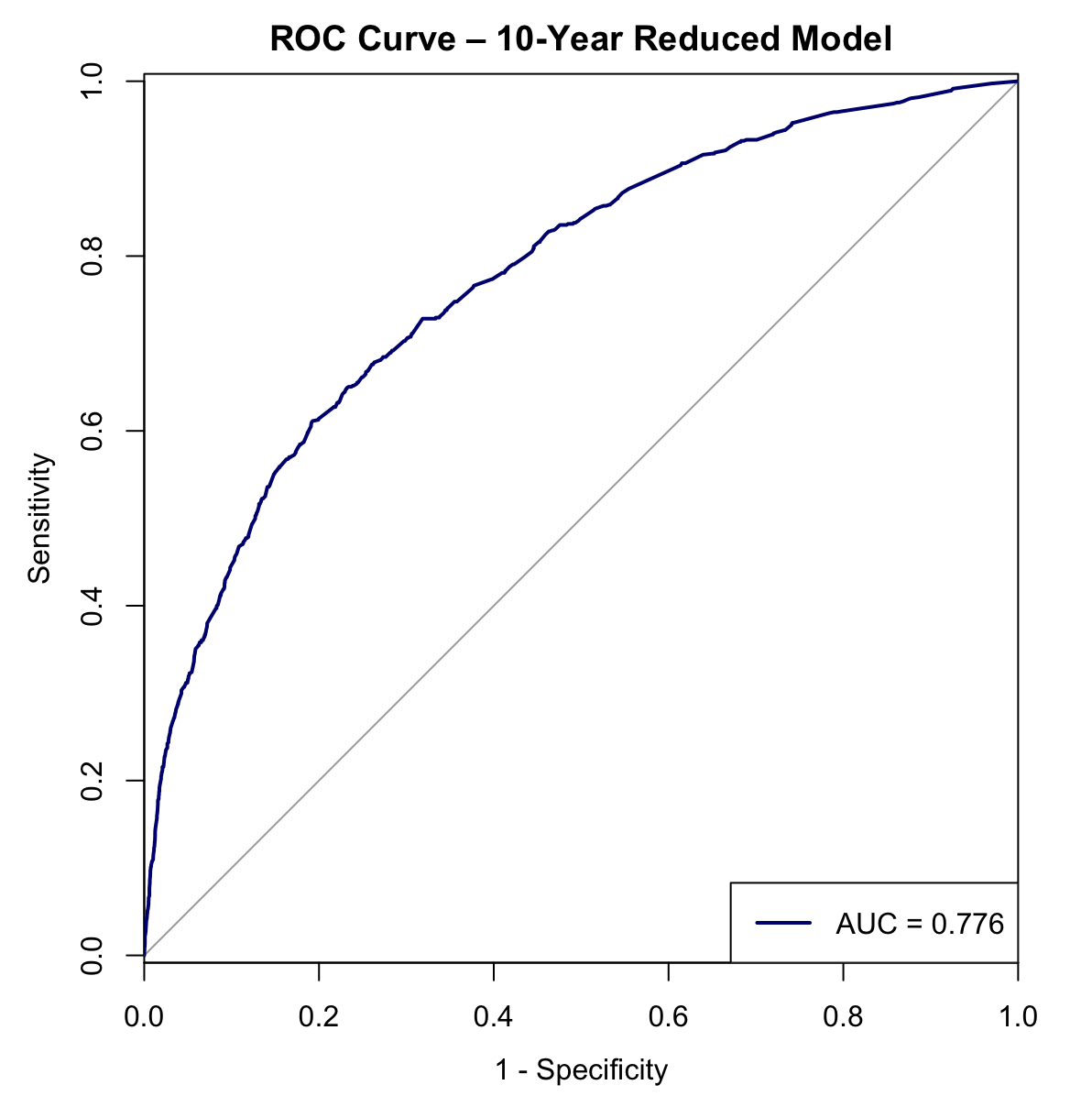
**

F**igure 3.** Calibration plots of the full versus reduced 10-year BCSM models


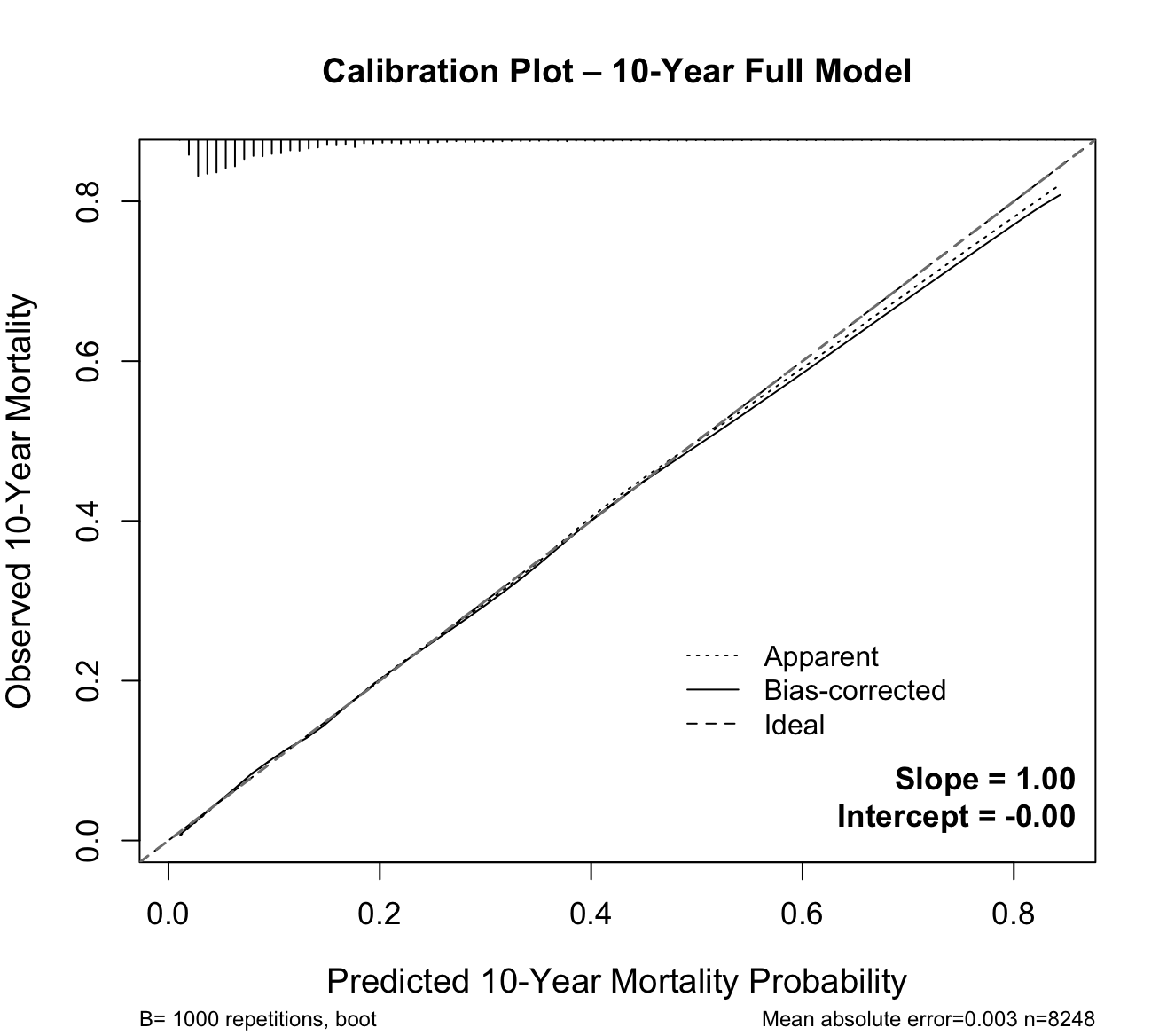


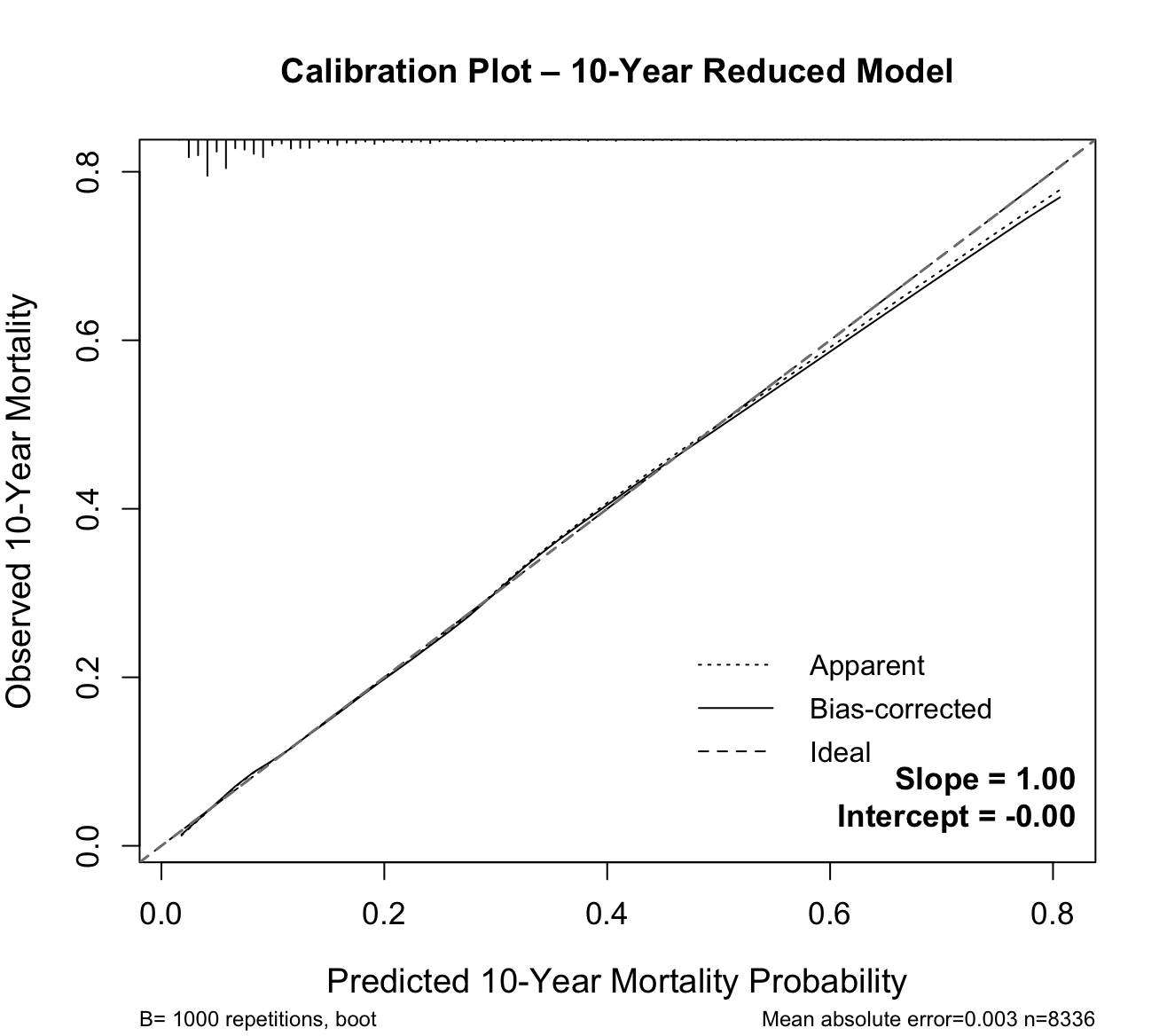


**Figure 4**: Unadjusted versus full adjusted versus reduced adjusted hospital variation in 10-year BCSM

**
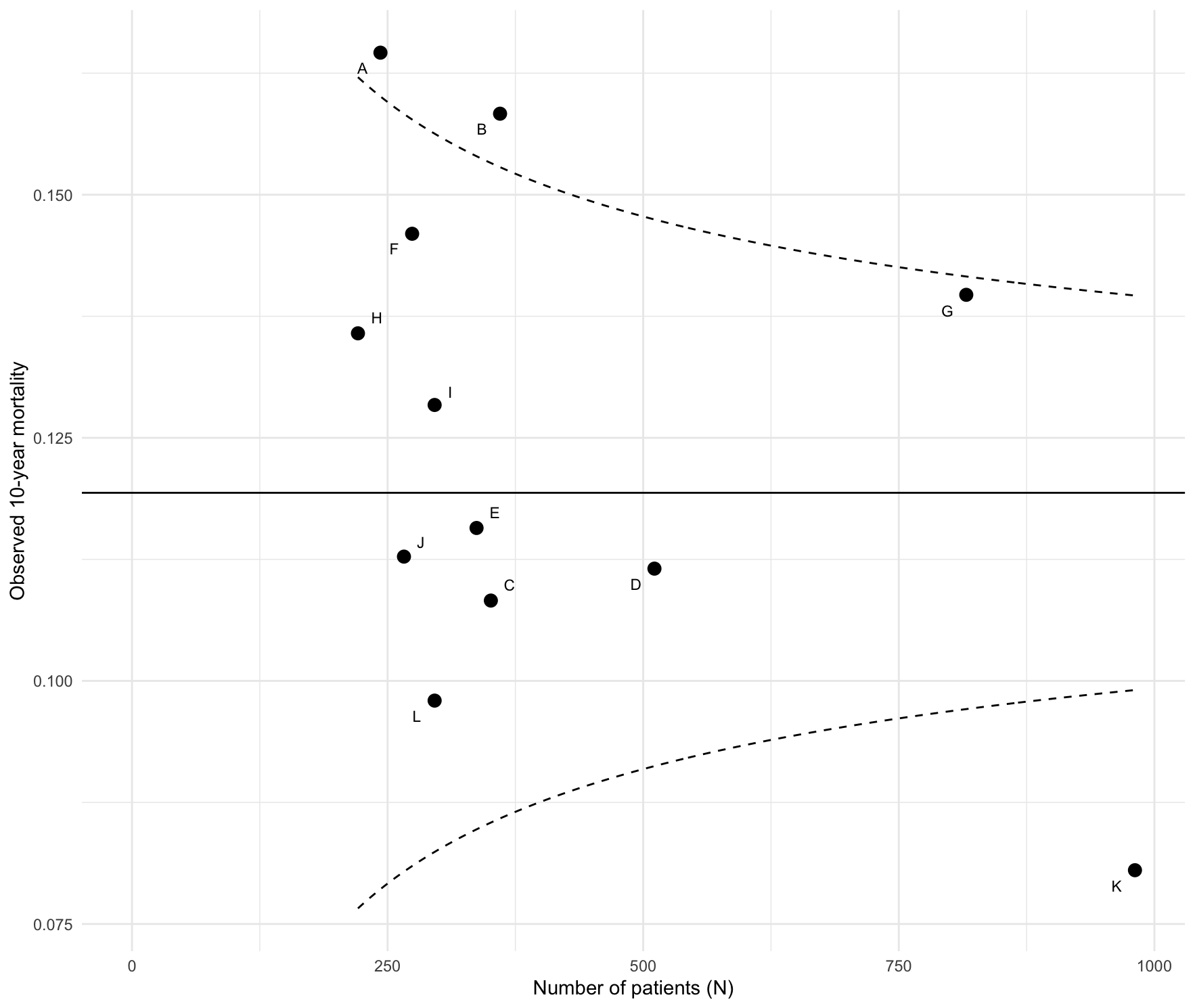
**

**Figure 5**: Adjusted hospital variation in 10-year BCSM (Full model)

**
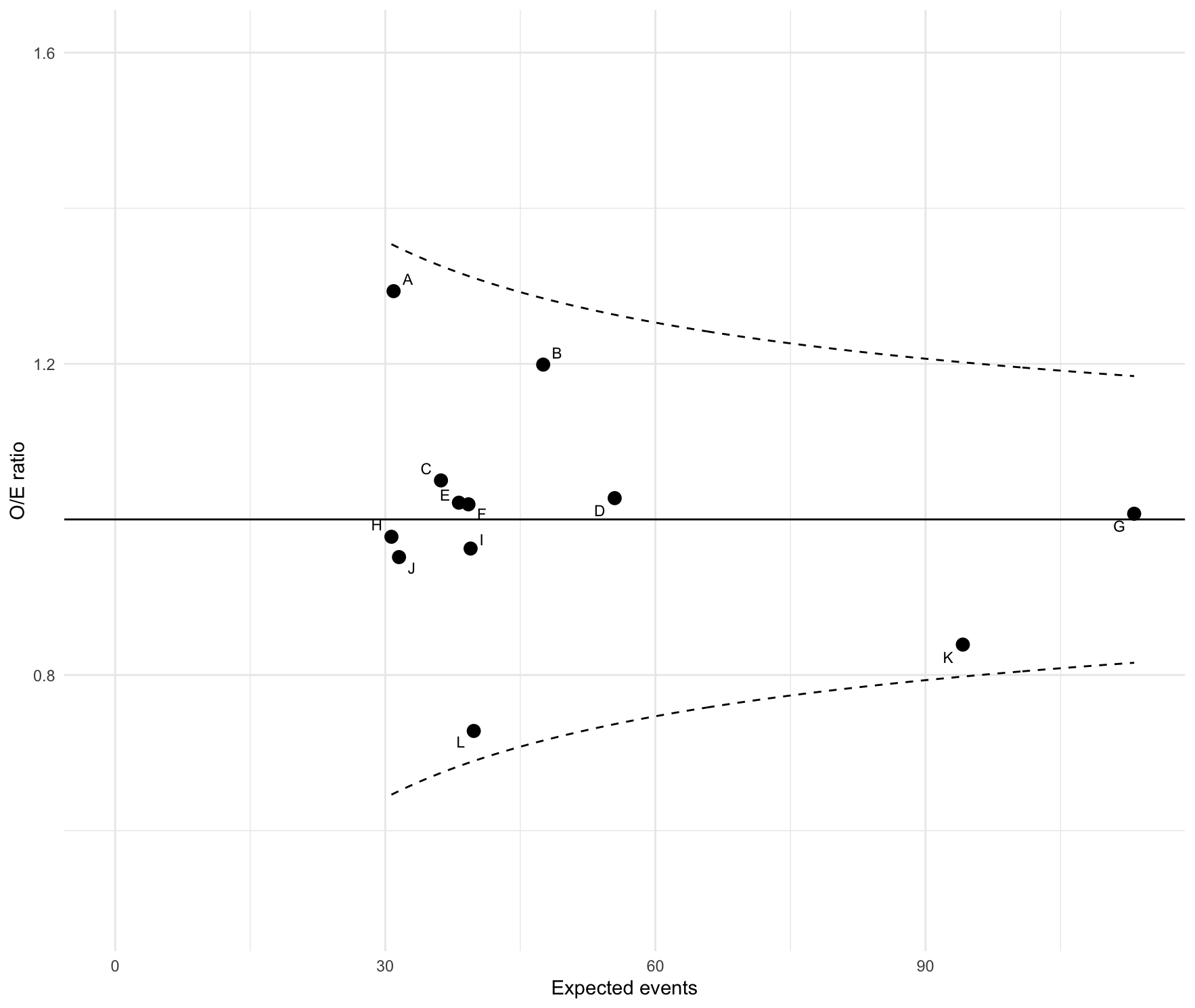
**

**Figure 6**: Adjusted hospital variation in 10-year BCSM (Reduced model)

**
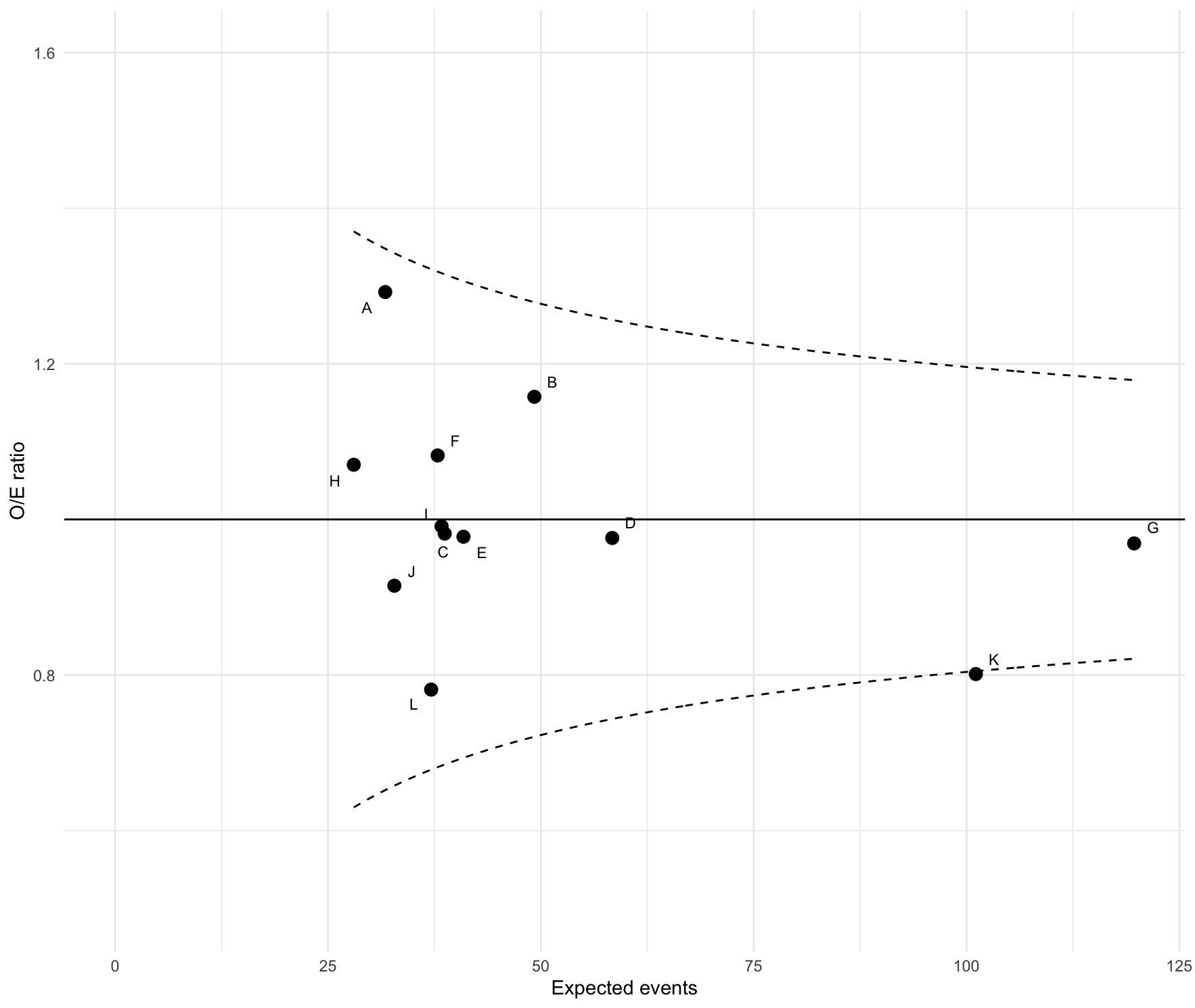
**

**
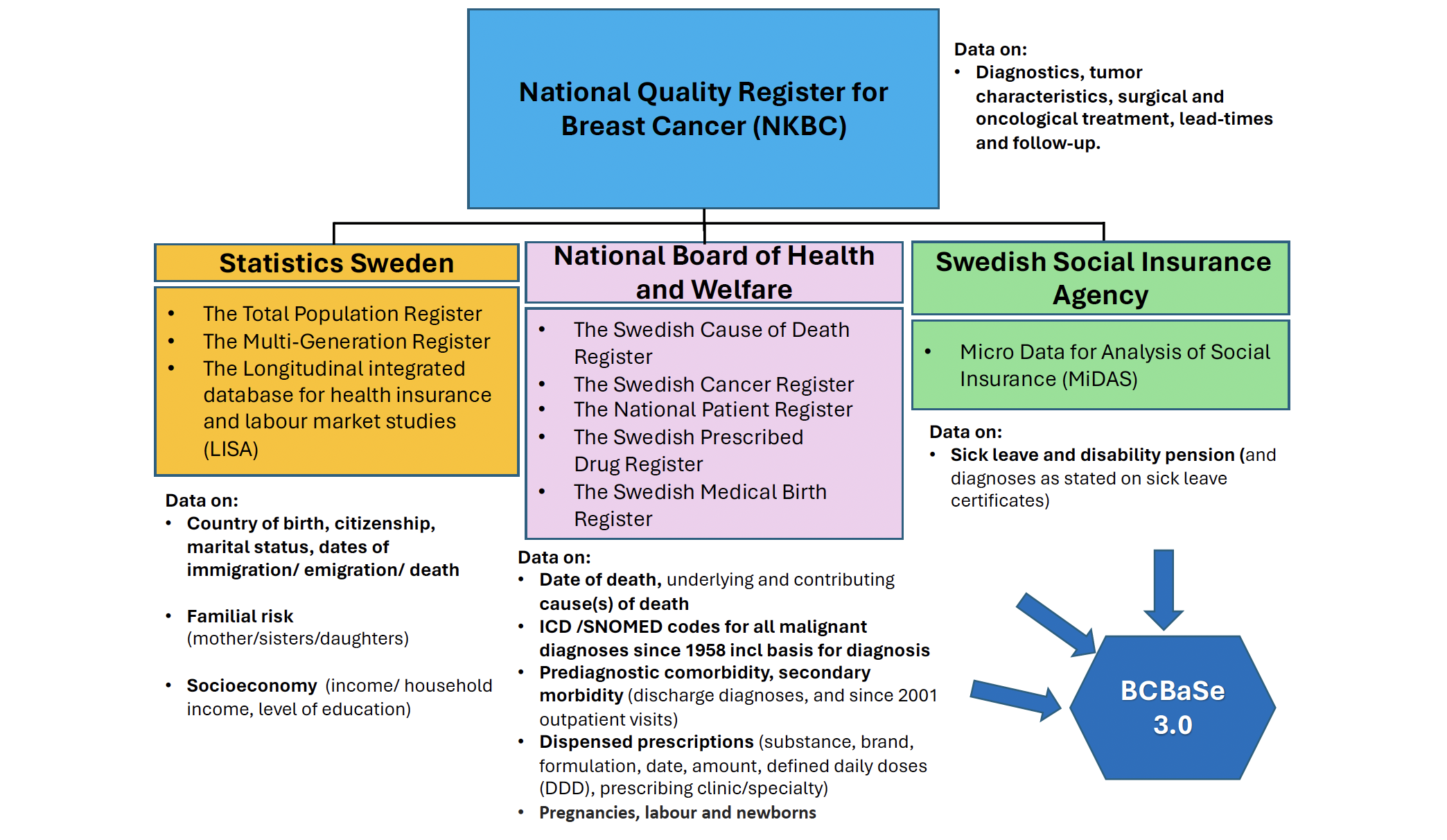
Supplementary Figure 1.** Description of the BCBaSe 3.0

**Supplementary Figure 2.** Scatter plot illustrating the correlation between the full- and reduced models’ estimated 10-year BCSM O/E ratios per hospital


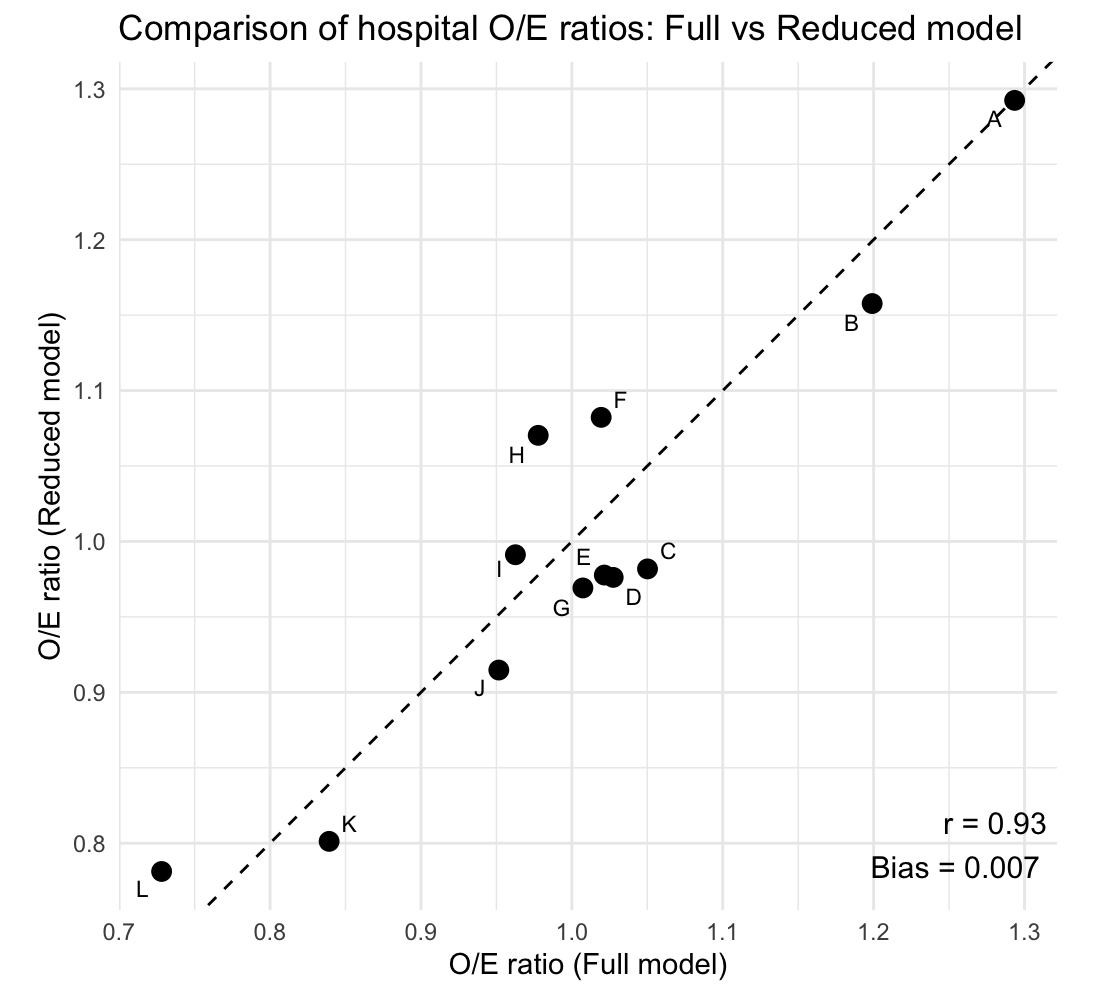


**Supplementary Figure 3.** Calibration plots for the 10-year BCSM full model in subtypes of BC


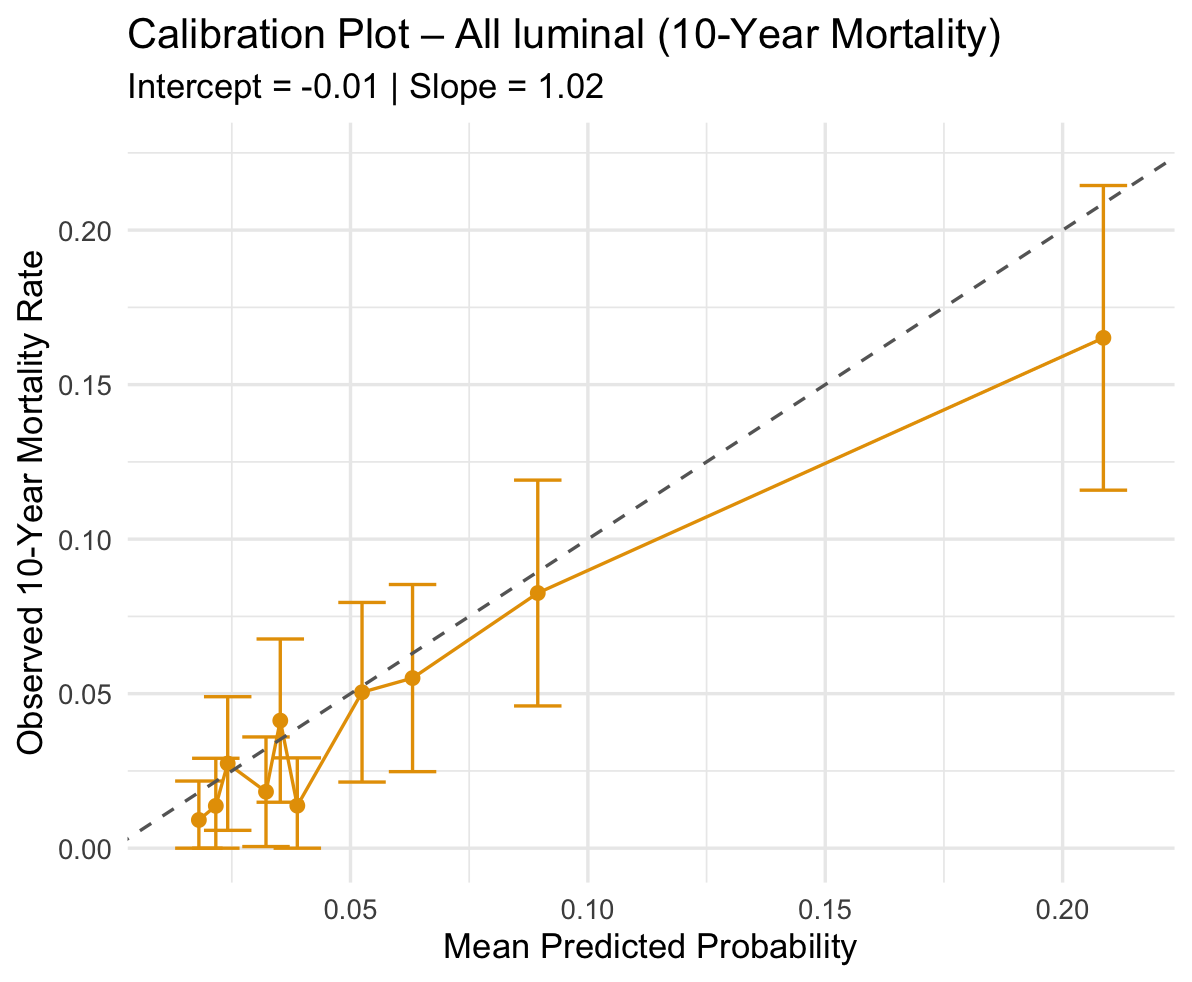

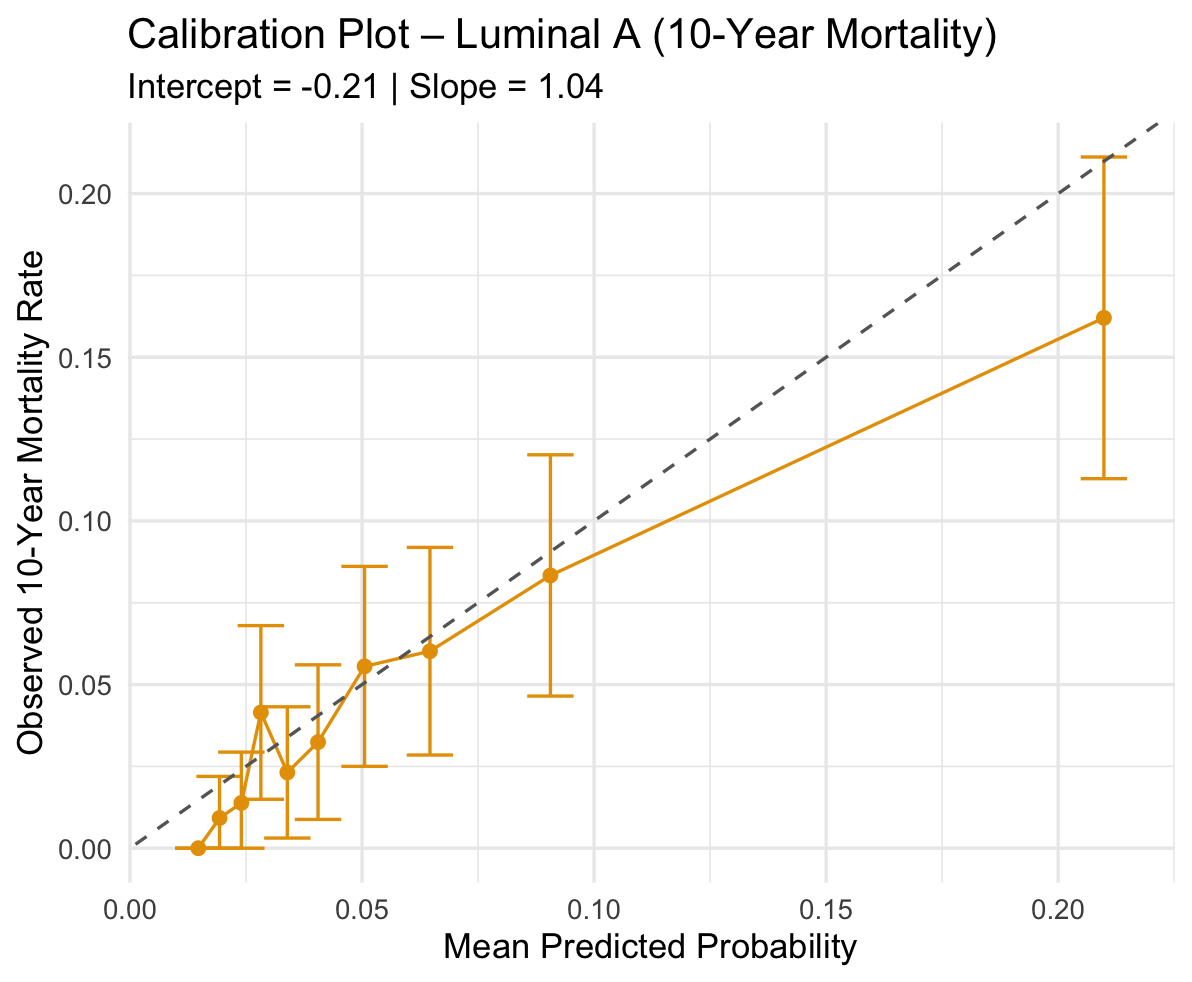

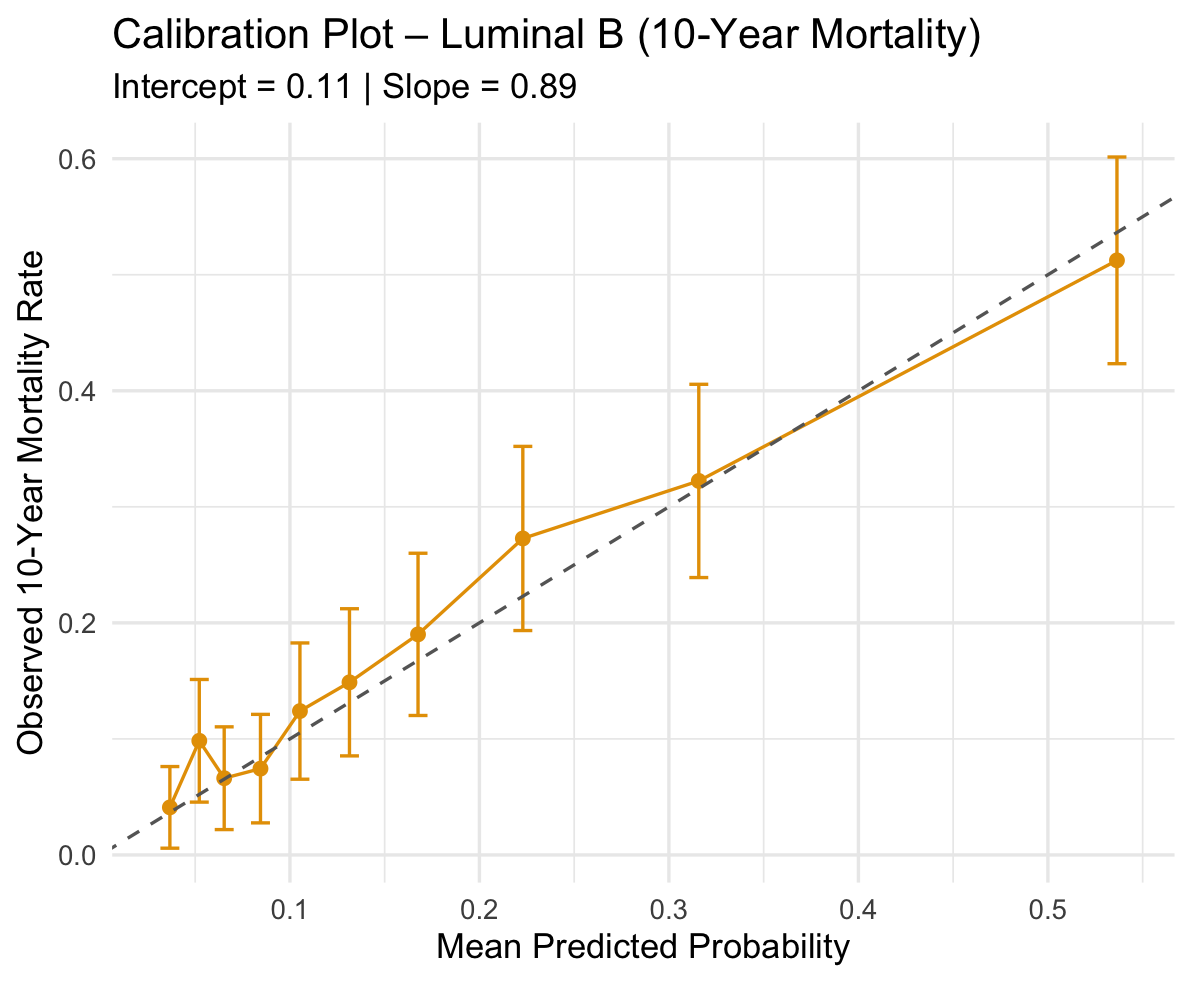

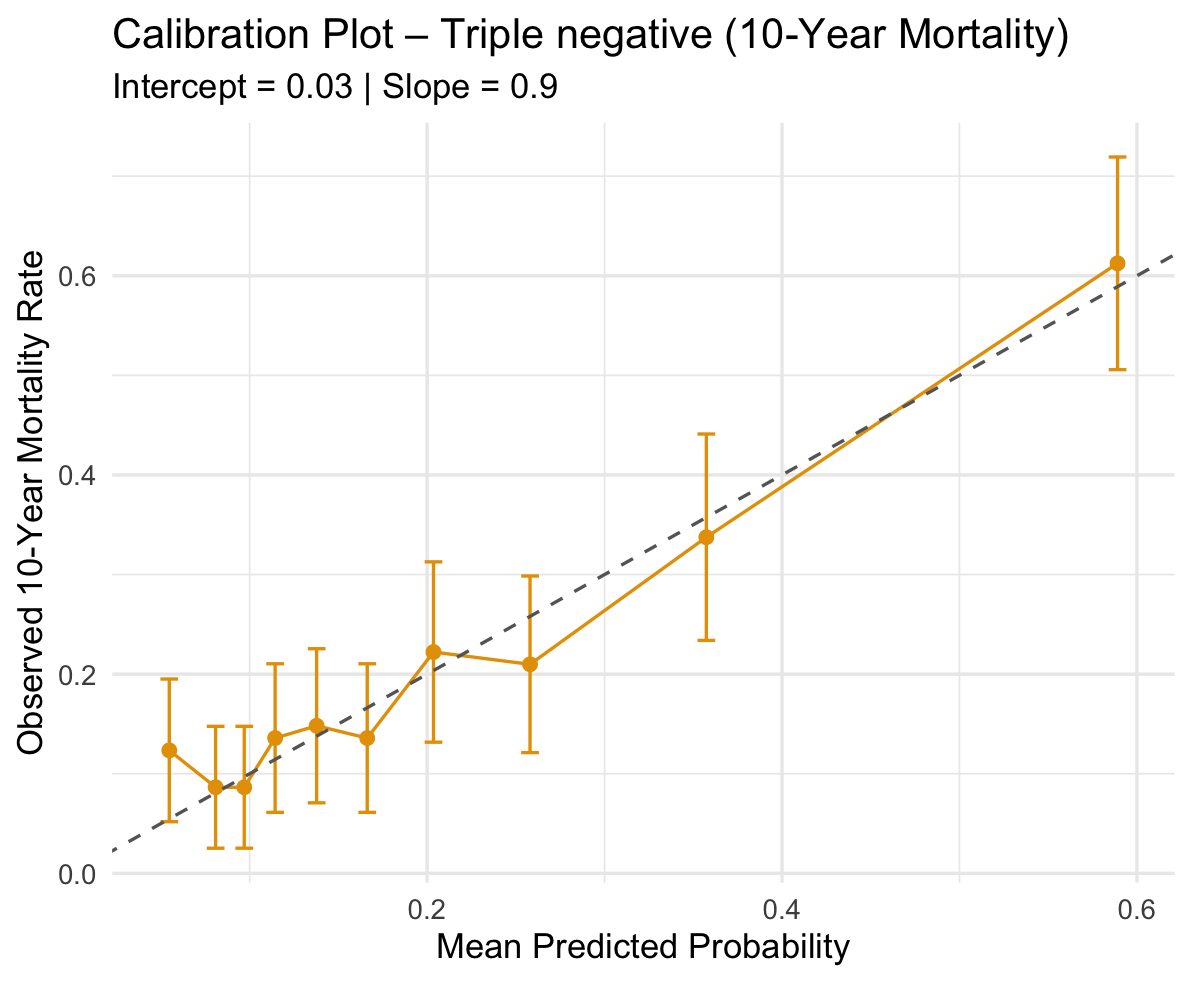


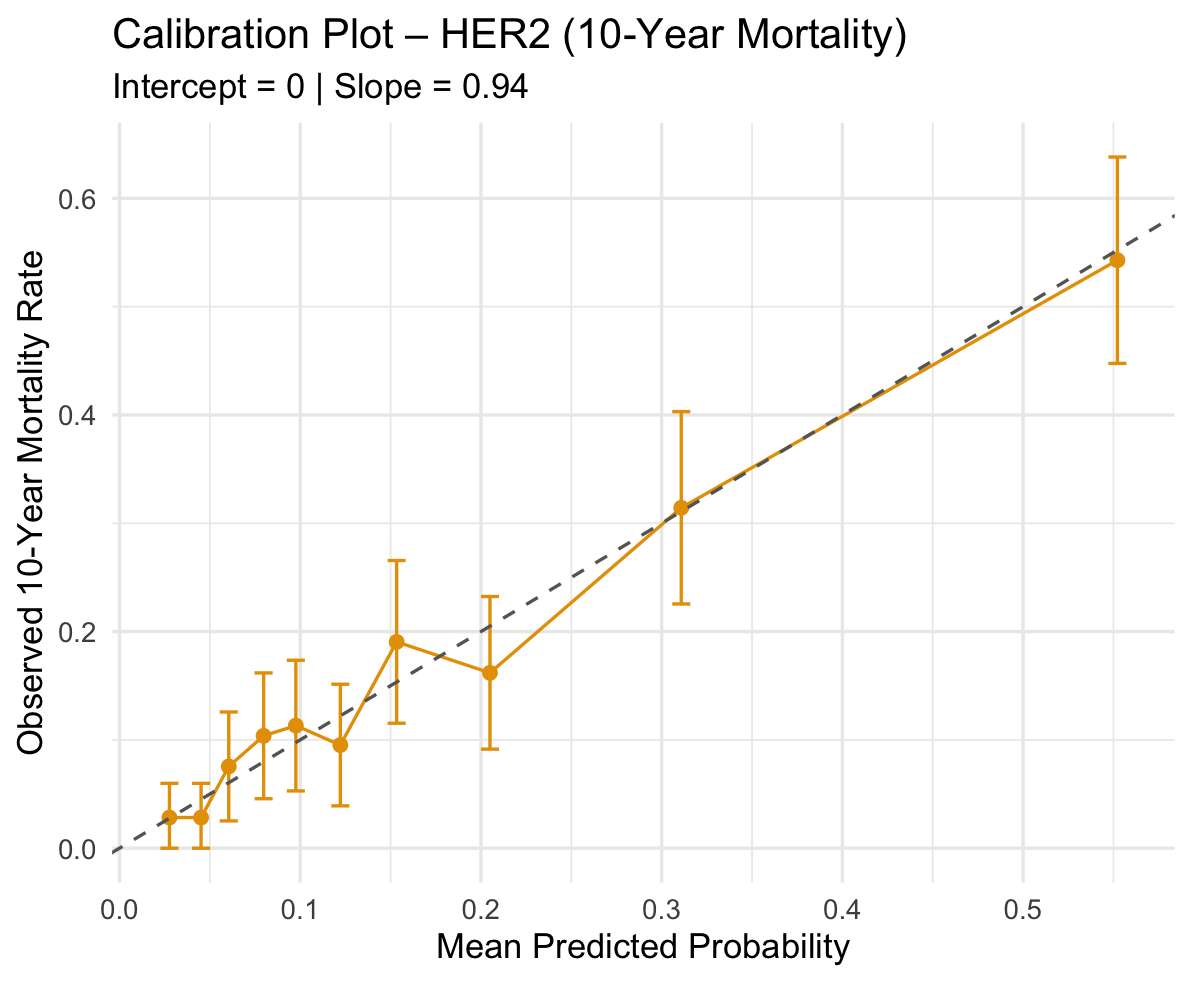


**Supplementary Figure 4.** Calibration plots for the 10-year BCSM reduced model in subtypes of BC


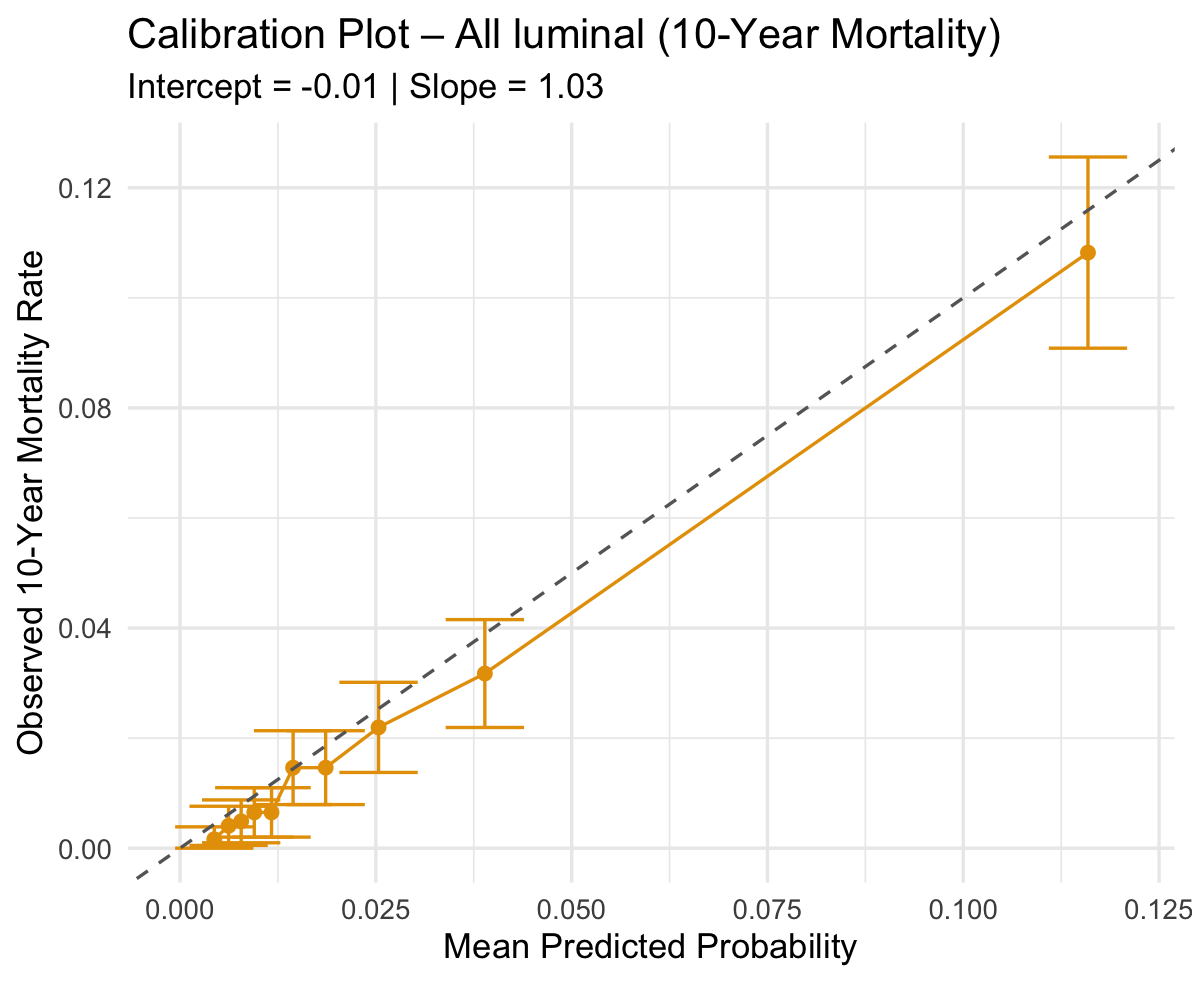

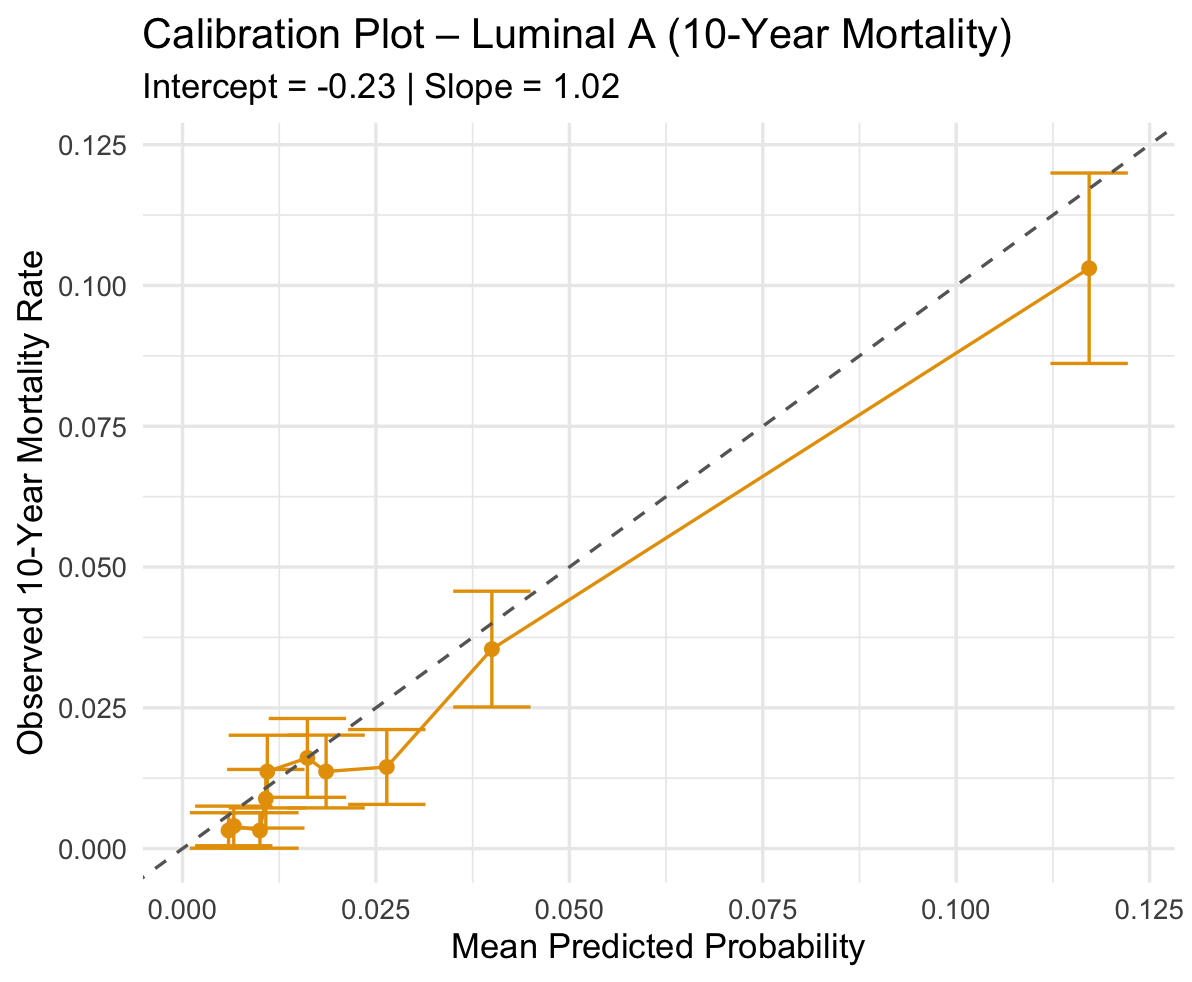

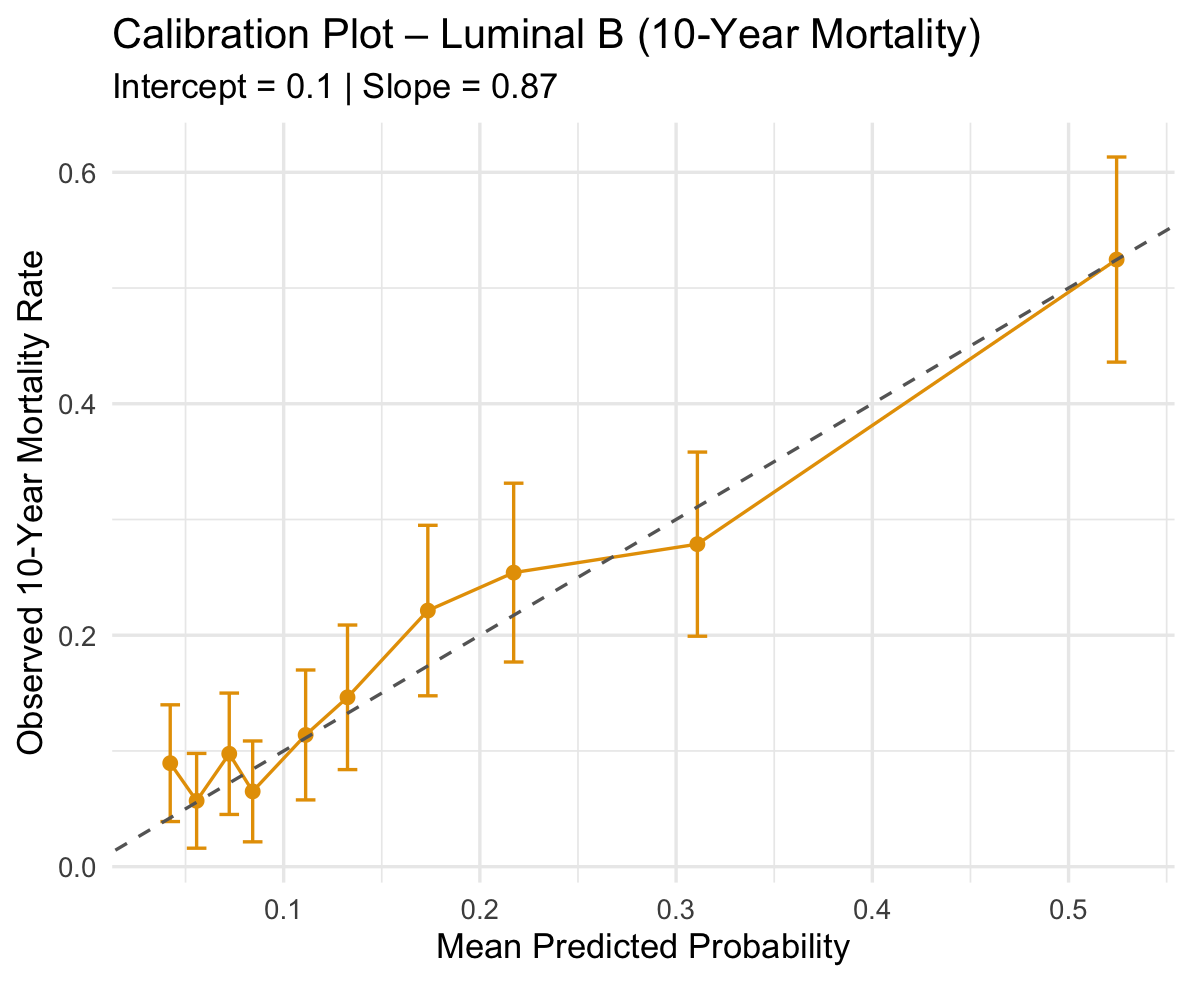

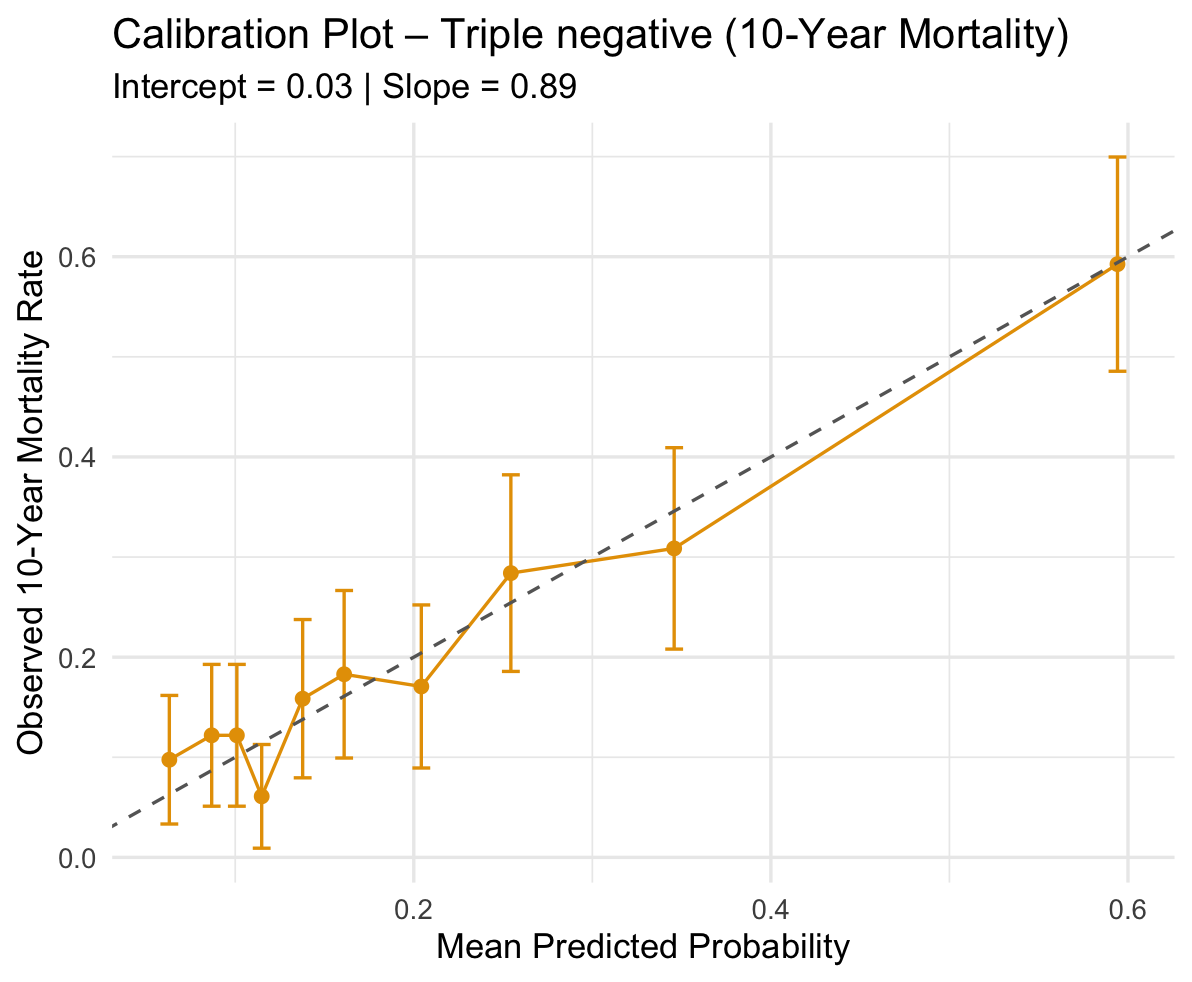


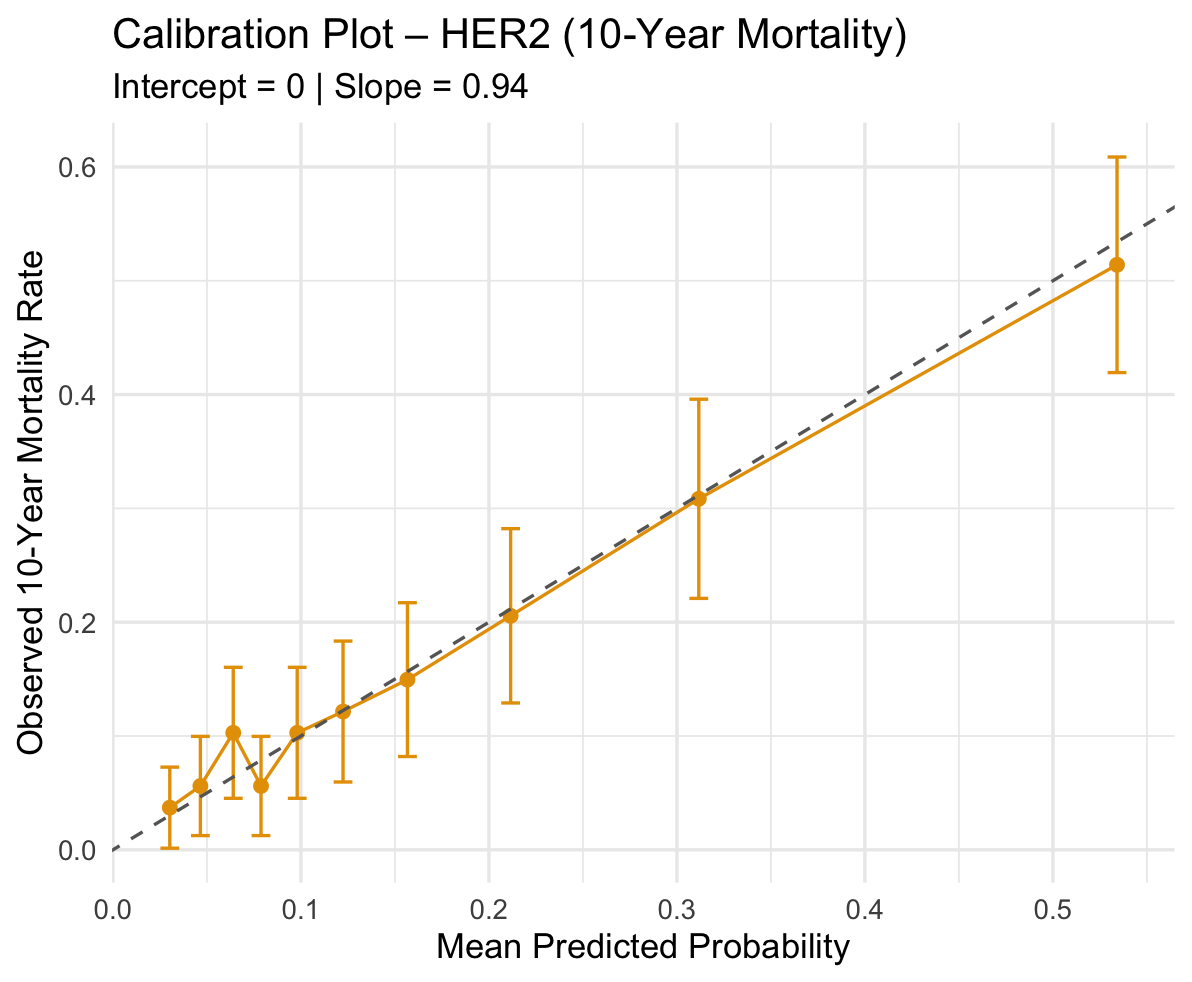


**Supplementary Figure 5.** Flowchart of included patients in the 5-year BCSM model


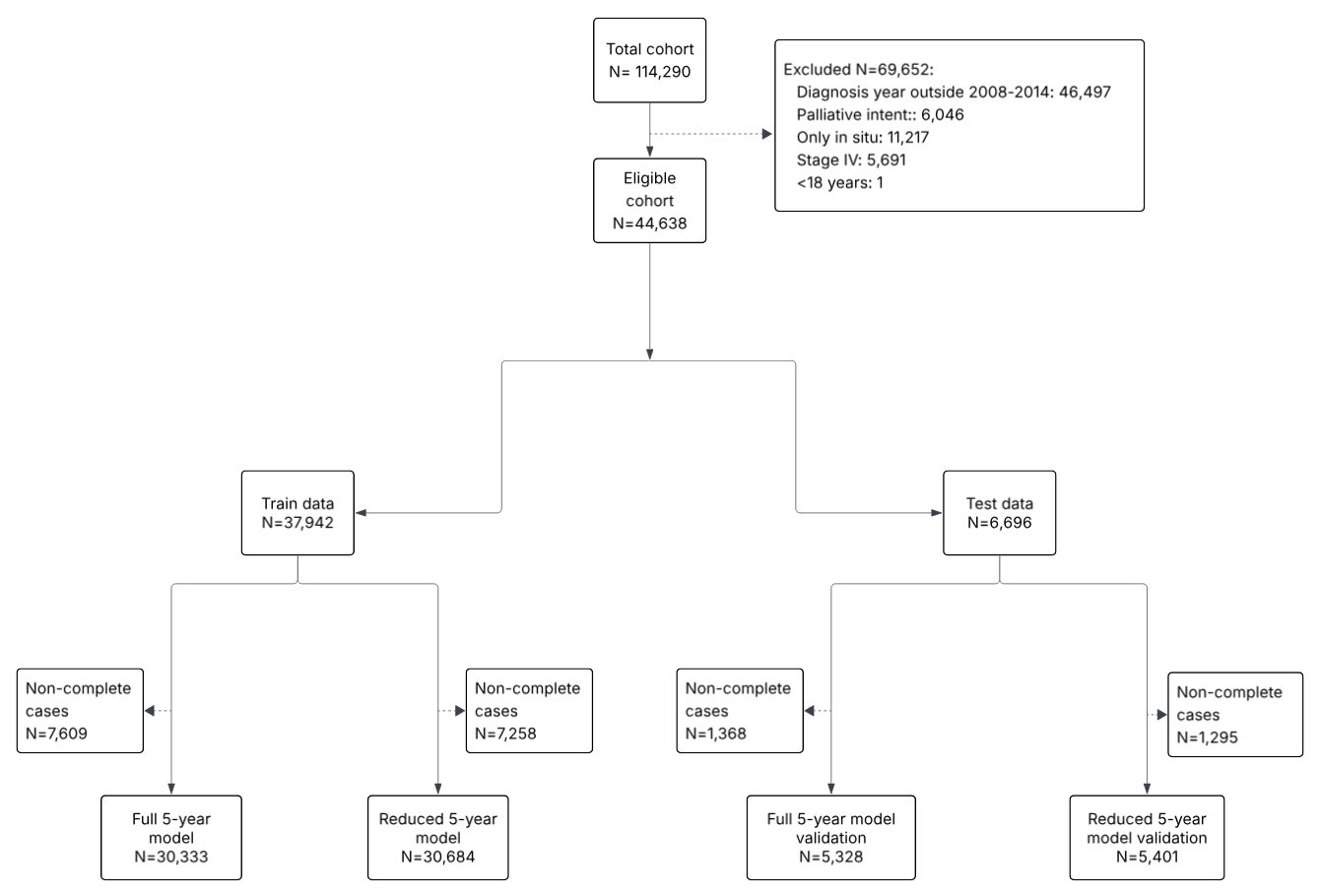


**Supplementary Figure 6.** AUC plots of the full versus reduced 5-year BCSM models

**
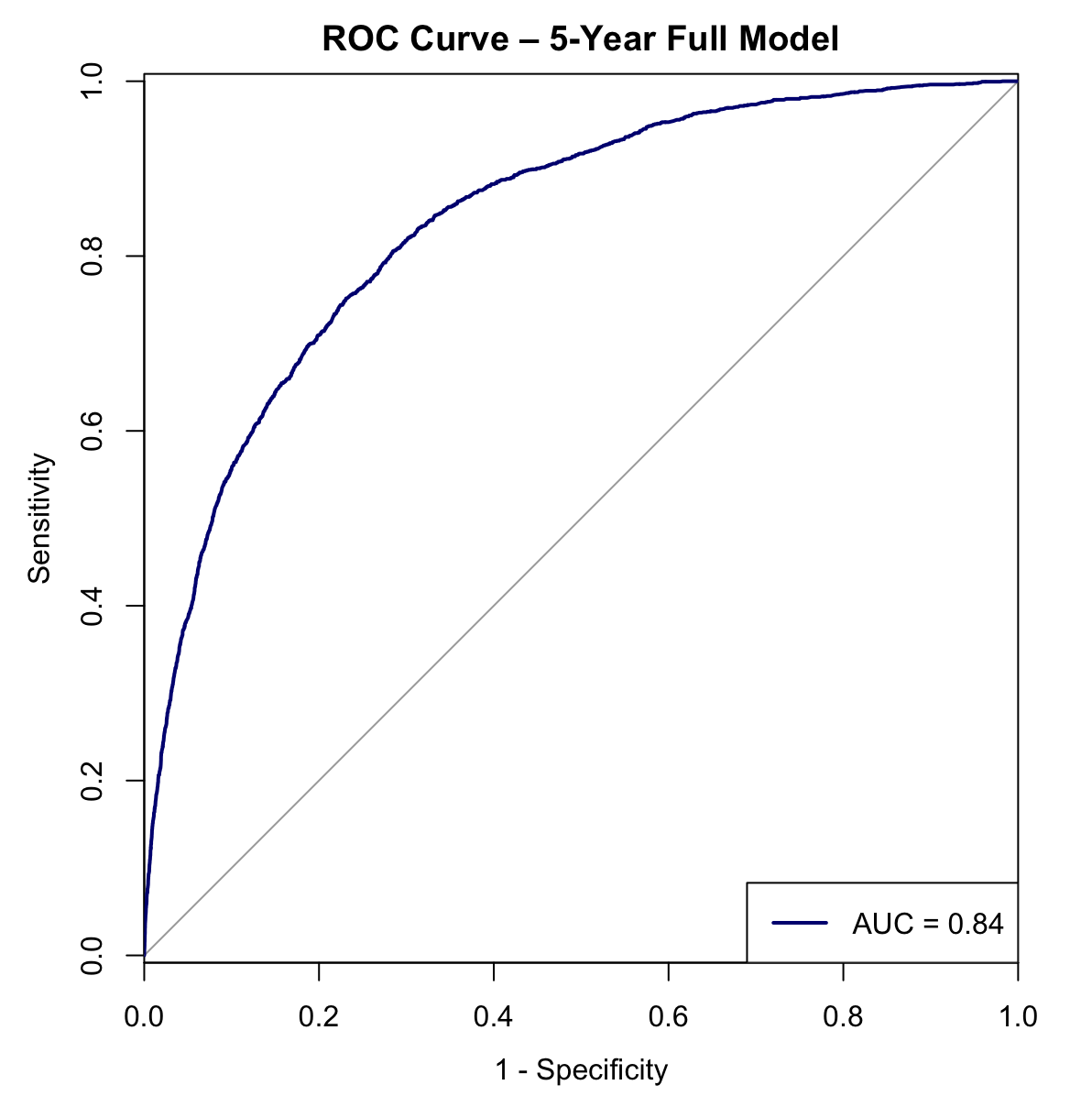
**


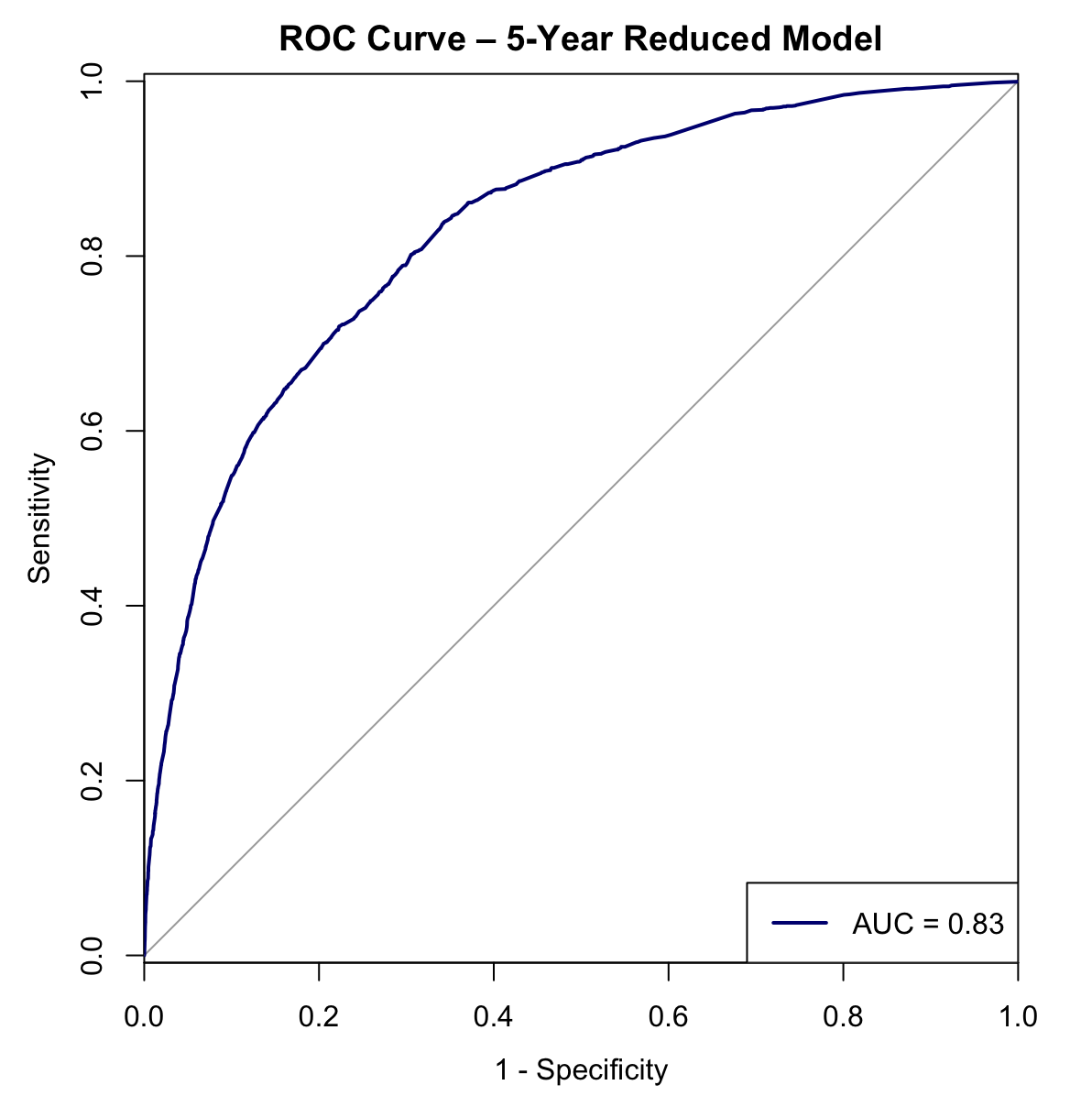


**Supplementary** **Figure 7.** Calibration plots of the full versus reduced 5-year BCSM models


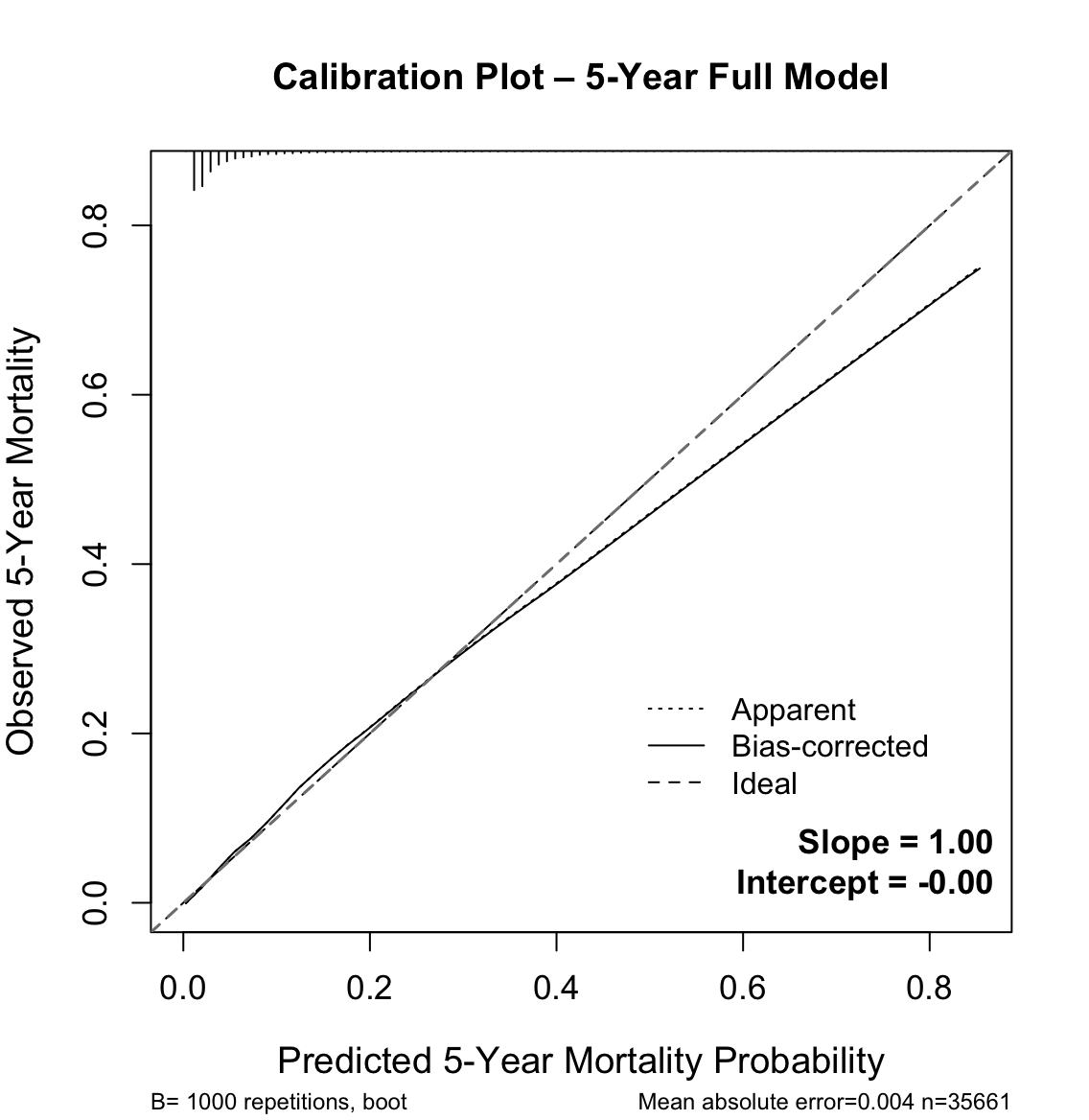


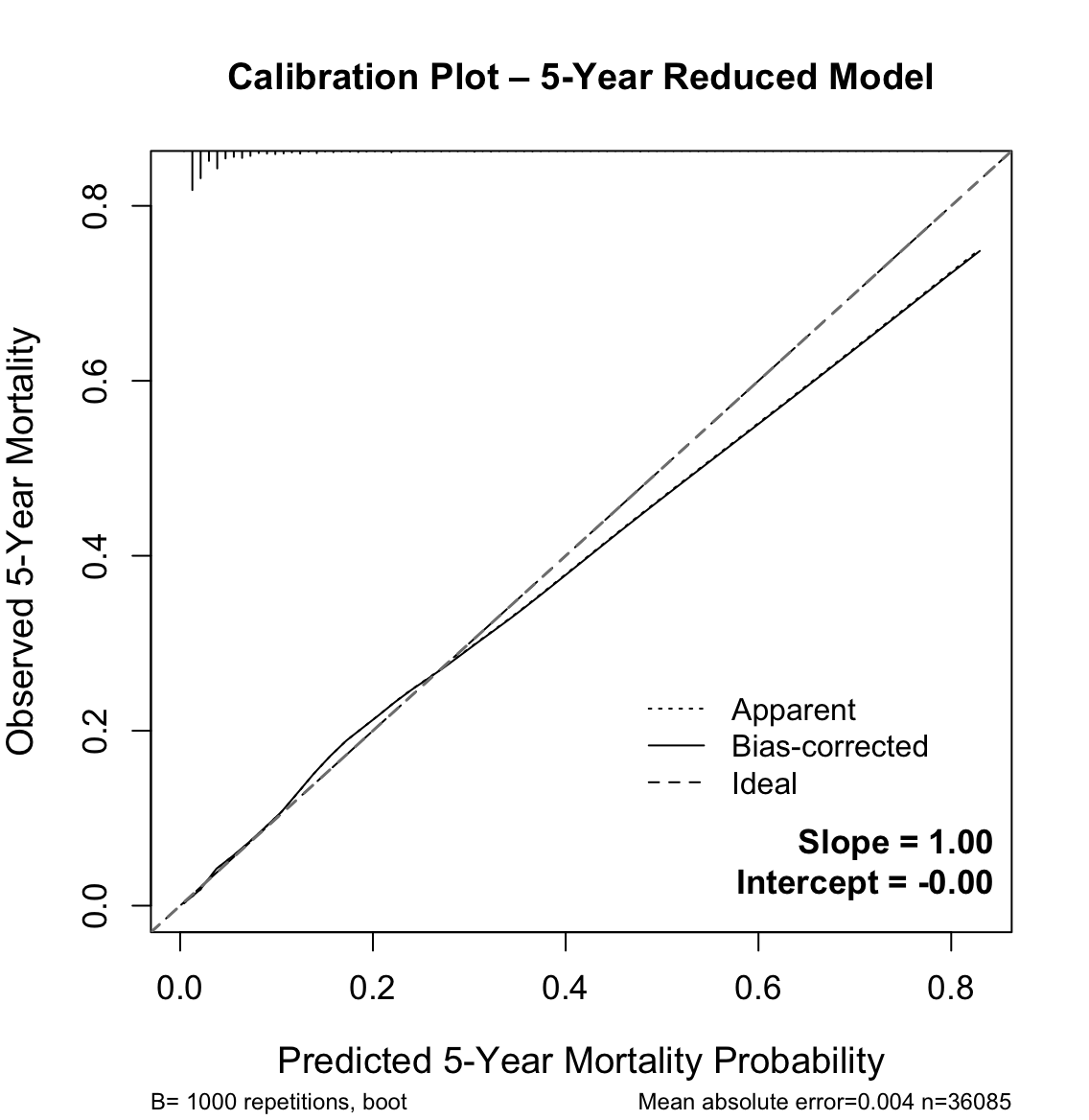


**Supplementary Figure 8**: Unadjusted hospital variation in 5-year BCSM

**
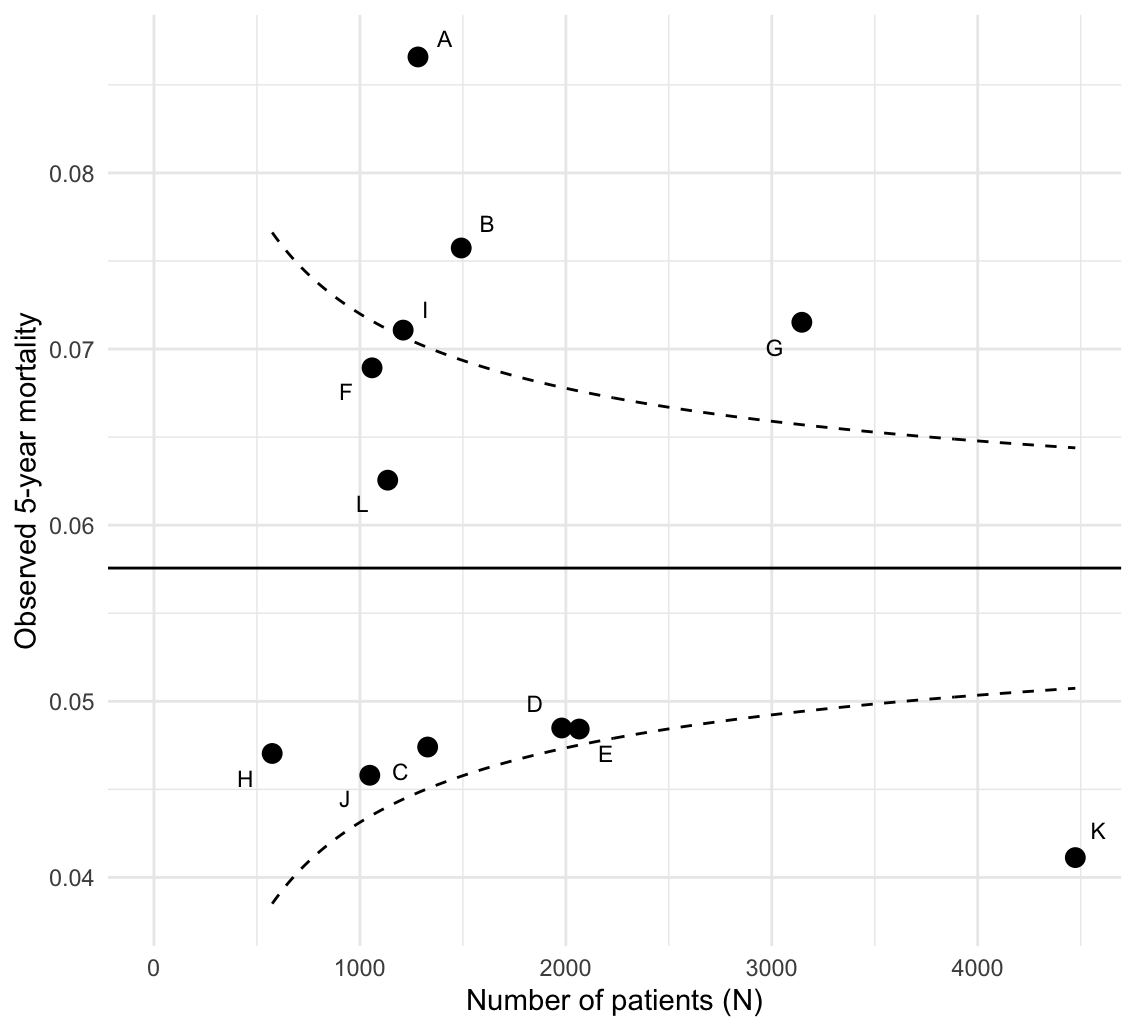
**

**Supplementary Figure 9**: Adjusted hospital variation in 5-year BCSM (Full model)


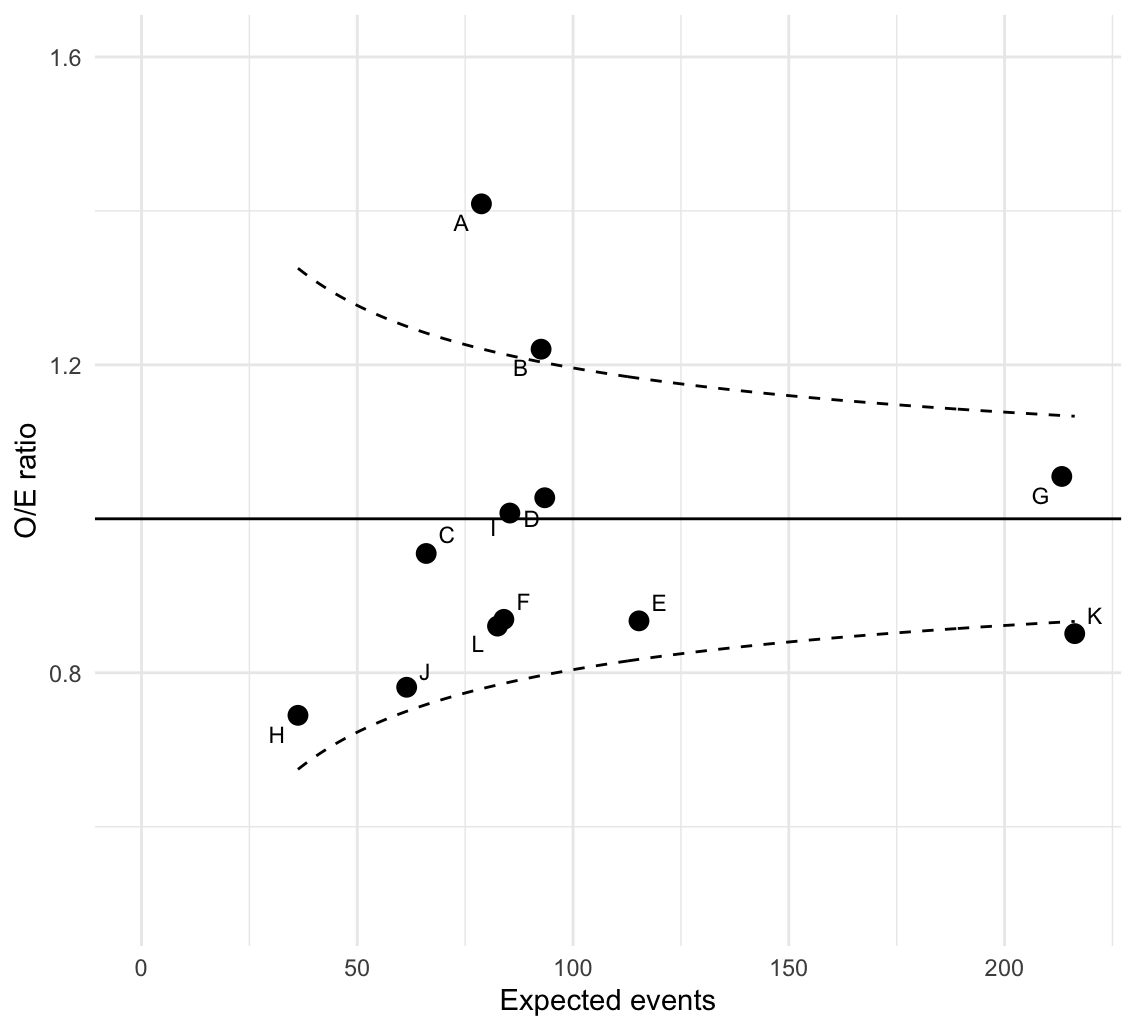


**Supplementary Figure 10**: Adjusted hospital variation in 5-year BCSM (Reduced model)


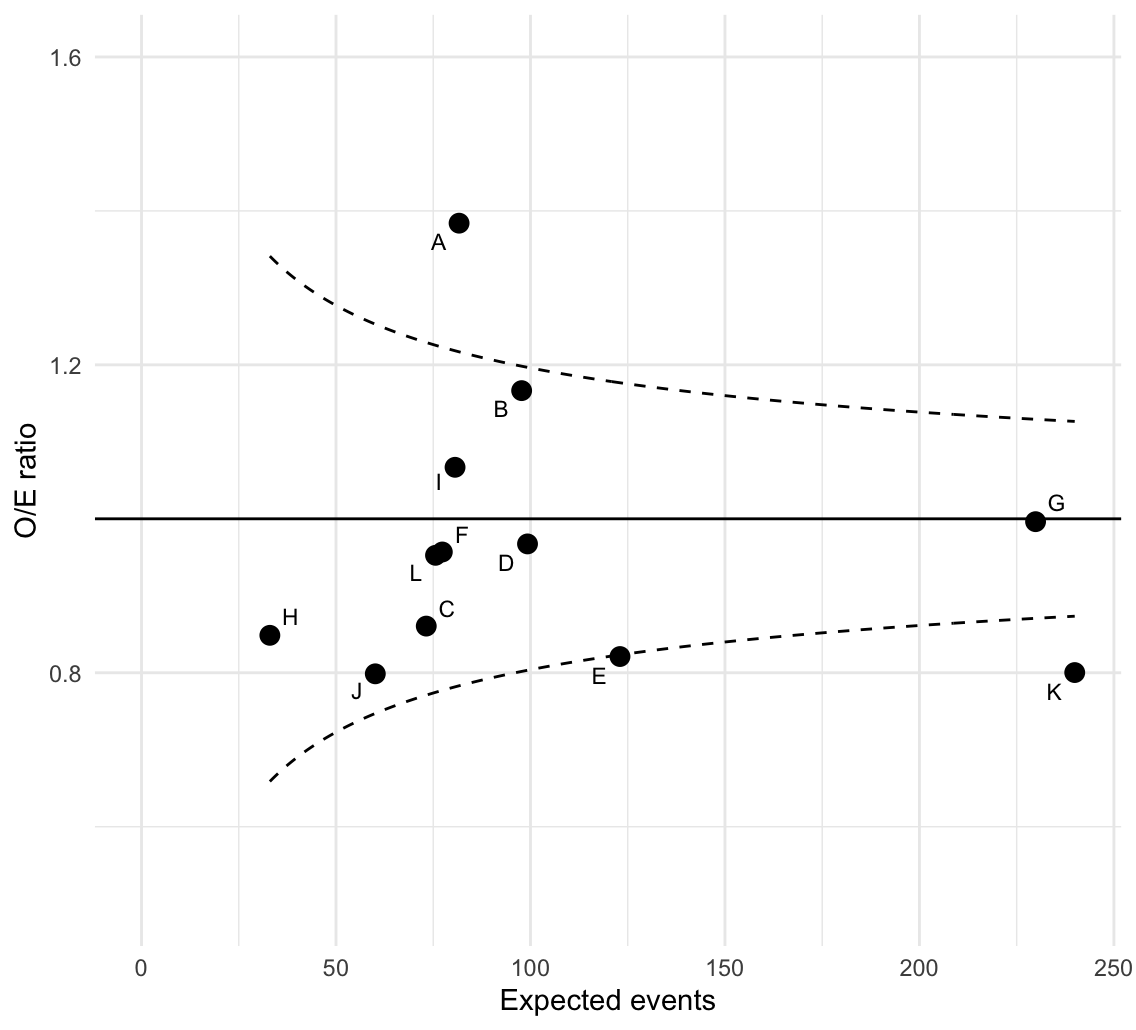

Supplement: Multimedia component 1 [file mmc1.docx]
